# Epigenetic Regulation of Cell Type–Specific Expression Patterns in the Human Mammary Epithelium

**DOI:** 10.1371/journal.pgen.1001369

**Published:** 2011-04-21

**Authors:** Reo Maruyama, Sibgat Choudhury, Adam Kowalczyk, Marina Bessarabova, Bryan Beresford-Smith, Thomas Conway, Antony Kaspi, Zhenhua Wu, Tatiana Nikolskaya, Vanessa F. Merino, Pang-Kuo Lo, X. Shirley Liu, Yuri Nikolsky, Saraswati Sukumar, Izhak Haviv, Kornelia Polyak

**Affiliations:** 1 Departments of Medical Oncology, Dana-Farber Cancer Institute, Boston, Massachusetts, United States of America; 2 Department of Medicine, Brigham and Women's Hospital, Boston, Massachusetts, United States of America; 3 Department of Medicine, Harvard Medical School, Boston, Massachusetts, United States of America; 4 NICTA Victoria Research Laboratory, The University of Melbourne, Melbourne, Victoria, Australia; 5 Department of Electrical and Electronic Engineering, The University of Melbourne, Melbourne, Victoria, Australia; 6 Thomson Reuters, Healthcare and Science, Encinitas, California, United States of America; 7 Department of Computer Science and Software Engineering, The University of Melbourne, Melbourne, Victoria, Australia; 8 Bioinformatics and System Integration, Baker IDI Heart and Diabetes Institute, Melbourne, Victoria, Australia; 9 Department of Biostatistics and Computational Biology, Dana-Farber Cancer Institute, Boston, Massachusetts, United States of America; 10 Harvard School of Public Health, Boston, Massachusetts, United States of America; 11 Department of Oncology, Johns Hopkins University School of Medicine, Baltimore, Maryland, United States of America; 12 Department of Biochemistry, The University of Melbourne, Melbourne, Victoria, Australia; 13 Metastasis Research Lab, Peter MacCallum Cancer Centre, Melbourne, Victoria, Australia; 14 Harvard Stem Cell Institute, Cambridge, Massachusetts, United States of America; Friedrich Miescher Institute for Biomedical Research, Switzerland

## Abstract

Differentiation is an epigenetic program that involves the gradual loss of pluripotency and acquisition of cell type–specific features. Understanding these processes requires genome-wide analysis of epigenetic and gene expression profiles, which have been challenging in primary tissue samples due to limited numbers of cells available. Here we describe the application of high-throughput sequencing technology for profiling histone and DNA methylation, as well as gene expression patterns of normal human mammary progenitor-enriched and luminal lineage-committed cells. We observed significant differences in histone H3 lysine 27 tri-methylation (H3K27me3) enrichment and DNA methylation of genes expressed in a cell type–specific manner, suggesting their regulation by epigenetic mechanisms and a dynamic interplay between the two processes that together define developmental potential. The technologies we developed and the epigenetically regulated genes we identified will accelerate the characterization of primary cell epigenomes and the dissection of human mammary epithelial lineage-commitment and luminal differentiation.

## Author Summary

Cellular differentiation is a precisely controlled and largely irreversible process orchestrated by cell type–specific epigenetic programs. Abnormalities in these programs lead to developmental disorders and play a key role in tumorigenesis. To better understand the regulation of human mammary epithelial cell type specification, we analyzed the gene expression, DNA methylation, and histone H3 K4 and K27 trimethylation profiles of progenitor-enriched and more differentiated luminal epithelial cell populations from multiple individuals. Network analysis of these profiles and their comparison to that of human embryonic stem cells identified key regulators of mammary epithelial and luminal lineage commitment. The list of genes epigenetically regulated in a cell type–specific manner provides a rich resource for the further analysis of human breast development and the role of epigenetic mechanisms in breast tumorigenesis.

## Introduction

Cellular differentiation is a well-orchestrated epigenetic program by which the developmental potential of the cells is progressively restricted. In adult tissues reversal of such programs is rarely observed with the exception of tissue regeneration, metaplasia, and neoplastic transformation. However, with the iPS (induced pluripotent stem cells) technology, directed cellular reprogramming is becoming a reality with wide implications in human disease [1]. The successful application of this technology requires the accurate understanding of cell type–specific epigenetic regulatory programs that depend on DNA methylation, chromatin (histone) modification, and non-coding RNAs. Each of these mechanisms has been shown to play a role in regulating stem cell function and differentiation, as well as tumorigenesis and they have been extensively studied in embryonic stem cells (ESCs) [2]–[4]. However, the genome-wide chromatin and DNA methylation patterns of human adult tissue-specific stem cells (ASCs) have not been explored.

In the normal human breast, the cellular identity and molecular characteristics of mammary epithelial stem cells have not been defined. A bipotential mammary epithelial stem cell thought to give rise to the two major cell types of the mammary duct: luminal epithelial and myoepithelial cells. Using various cell culture and xenotransplant assays, several candidate progenitors have been identified and numerous cell-surface markers [5]–[12], mammosphere cultures [8], and ALDH enzyme activity assay [13] have been proposed to enable their enrichment. Among others, lineage^-^/CD24^-/low^/CD44^+^ (“CD44+”) cells were found to contain cells with stem cell properties based on clonogenicity assays in cell culture and mammary fat pad transplantation assays in mice [13]–[15]. To characterize more differentiated luminal CD24+ and progenitor-enriched CD44+ breast epithelial cells at the molecular level, we isolated these cells from normal breast tissue and analyzed their comprehensive gene expression profiles and clonogenicity [16], [17]. We determined that the functional properties and gene expression patterns of CD24+ and CD44+ cells were consistent with the hypothesis that they represent luminal lineage-committed and progenitor-enriched cells, respectively. Furthermore, the expression profiles of these cells displayed high similarity to progenitor and luminal-restricted fractions isolated using other markers such as EpCam and CD49f [11], [12], [18].

Although the CD44+ and CD24+ cell fractions are not homogenously pure, they represent more progenitor and more differentiated luminal epithelial cell states. Thus, to begin dissecting the regulation of mammary epithelial and luminal lineage commitment, here we analyzed the genome-wide histone and gene expression and comprehensive DNA methylation profiles of CD44+ and CD24+ cells purified from normal human breast tissue samples. We found significant differences in the regulation of cell type–specific gene expression by histone and DNA methylation and identified candidate key transcriptional determinants of mammary epithelial and luminal cell lineages.

## Results

### Optimization of ChIP-Seq and other procedures for small cell numbers and Illumina platform

Prior studies have characterized the global histone modification, DNA methylation, and gene expression profiles of cultured cells [2], [19], [20], but similar studies have not been conducted in uncultured cells due to technical limitations. ChIP (Chromatin Immunoprecipitation) experiments in particular require large numbers of cells, which are difficult to obtain from primary tissues [21]. To overcome these technical challenges, first, we optimized ChIP-Seq (ChIP combined with high-throughput sequencing) [21] to enable the global analysis of small numbers (1–3×10^5^) of cells isolated from human tissue samples (Figure S1A and Protocol S1). To ensure that our modified and standard ChIP-Seq generate the same quality data, we analyzed the histone H3K27me3 (K27) profiles of 9×10^4^ and 3.4×10^6^ MCF-7 cells generated using the two different procedures.

**Figure 1:**
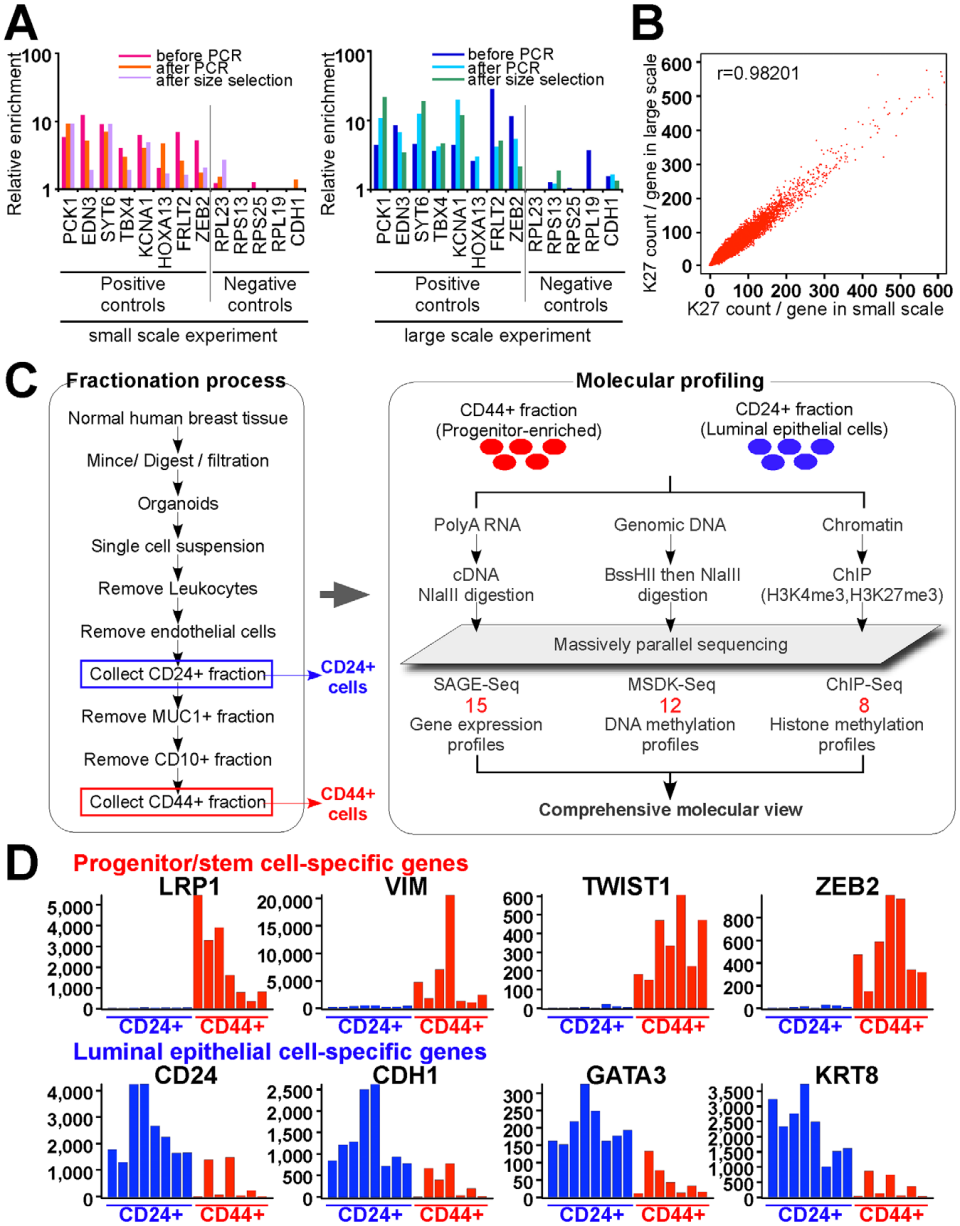
Overview of experimental design and data analysis. (A) qPCR validation steps during ChIP-Seq library preparation using small and large scale protocols. Several regions known to be K27-enriched (positive controls) or not-enriched (negative controls) in MCF-7 cells were tested by qPCR using DNA templates before and after linker-mediated PCR amplification and after size-selection. Y-axis indicates enrichment relative to averaged negative controls. (B) Comparison of small-scale and standard ChIP-Seq experiments. Scatter plots depict the counts of ChIP-Seq reads for each gene. X and Y-axes indicate mapped read counts around promoter regions (+/− 5 kb from TSS) for each gene in small-scale and in standard experiment, respectively. (C) Schematic view of cell purification and sample processing. Red numbers indicated the number of independent samples in each data type. (D) Representative examples of genes known to be specifically expressed in luminal (blue) and stem (red) cells. Each bar represents a different sample. Y-axis indicates SAGE-Seq tag counts.

Comparison of K27 enrichment of known positive and negative controls by quantitative PCR (qPCR) at critical steps during the process demonstrated excellent agreement between the two methods and also confirmed that the low-cycle PCR amplification required for sequencing using the Illumina Genome Analyzer does not alter the results (Figure 1A). Similarly, genome-wide comparison of K27 ChIP-Seq data generated using the two different methods demonstrated excellent agreement. For each sample we generated ∼20 million quality filtered reads, from which 65–70% could be uniquely aligned to the human genome (Protocol S1). Quantitative analyses confirmed strong concordance between the two datasets; the correlation coefficient was 0.98 (Figure 1B and Figure S1B). The percentage of duplicate reads were essentially the same (3.88 and 3.2% for small and large scale method, respectively), thus, amplification of lower amount of template does not generate PCR bias.

We also adapted MSDK (Methylation-Specific Digital Karyotyping) [22] and SAGE (Serial Analysis of Gene Expression) [23] protocols for the Illumina genome analyzer to enable the integrative analysis of all three types of data using the same platform. Using these newly developed approaches, we analyzed the histone H3K27me3 (K27) and H3K4me3 (K4) modification, DNA methylation, and gene expression profiles of CD44+ and CD24+ cells purified from normal human breast tissues (Figure 1C, all these data were deposited to GEO under accession # GSE26141). The expression of known stem cell (e.g., LRP1, ZEB2) [24], [25] and luminal epithelial cell-specific (e.g., GATA-3, CDH1) [26], [27] genes was consistently mutually exclusive in CD44+ and CD24+ cells, respectively, both by qPCR (Figure S2A) and SAGE-Seq (Figure 1D). Furthermore, the K27 enrichment of known positive and negative controls by qPCR displayed the expected patterns () validating the cell purification and profiling procedures.

### Genome-wide histone modification patterns and their functional relevance

**Figure 2:**
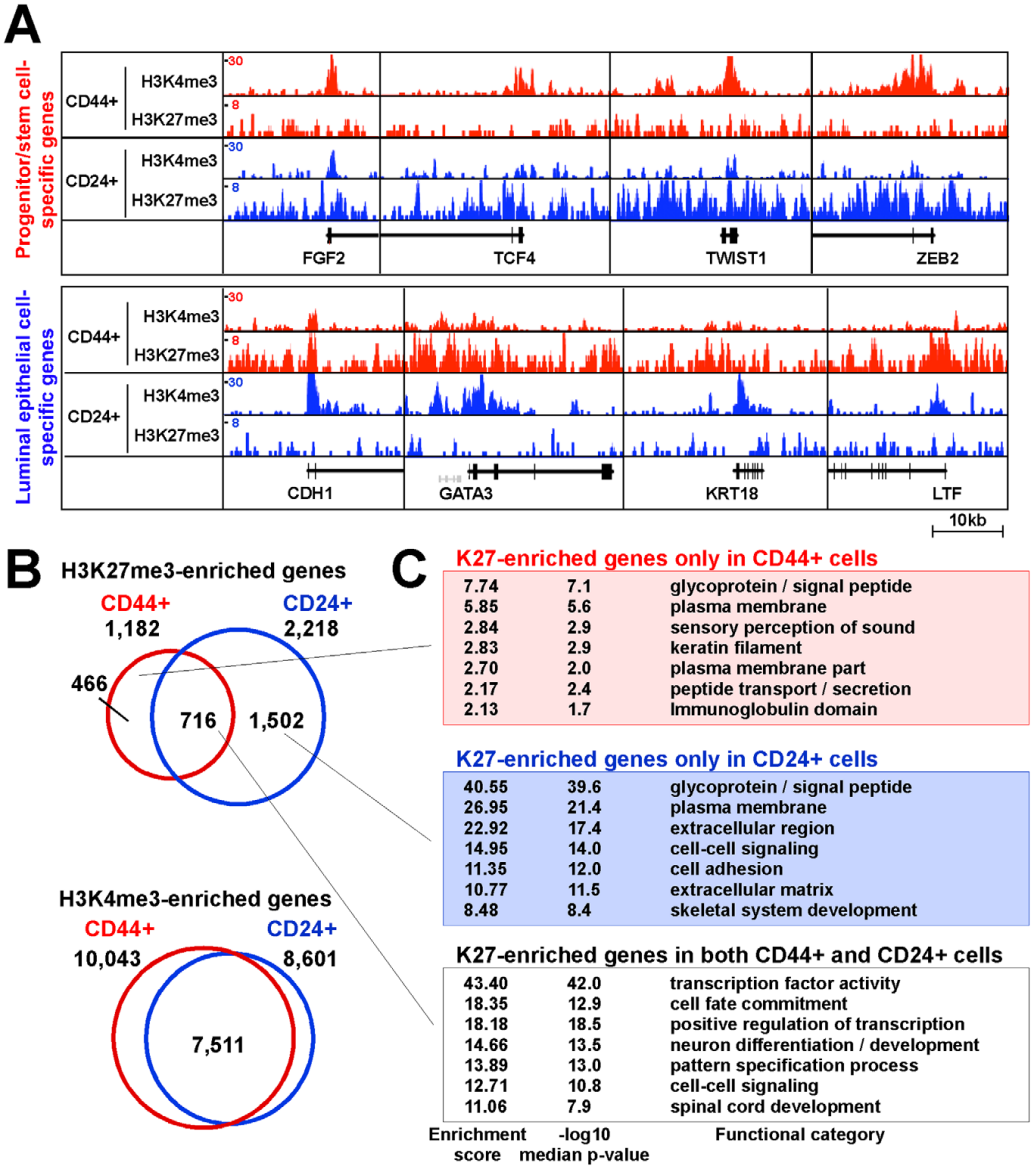
Distinct histone methylation profiles of human CD44+ mammary epithelial progenitors and CD24+ differentiated luminal epithelial cells. (A) Examples of histone modification patterns of genes known to be expressed in stem or luminal cells. Total aligned tag count in each ChIP-Seq library was scaled to 10 million, Y-axis shows tag counts averaged over a 10 bp window. (B) Comparison of genes enriched for the indicated histone marks in CD44+ and CD24+ cells. Venn diagram depicts the number of unique and overlapping genes. (C) Functional enrichment analysis of K27-enriched genes using DAVID Functional Annotation Tool.

We generated a total of eight ChIP-Seq libraries for K4 and K27 from CD44+ and CD24+ cells, four from one individual (sample 1) and four others from two individuals (samples 2 and 3). We mainly used data from sample 1 for follow up analyses as in this case both K4 and K27 data were available for both cell types. In addition, we also generated ChIP-Seq libraries using input DNA from each of the six cell populations analyzed and these were used as background to define K27 and K4 enriched regions. We mainly focused on K27-enriched genes due to the importance of PRC2 in ESCs [3], [4], [19], [20]. ChIP-Seq demonstrated clear differences of histone methylation patterns in the two cell types for known stem and luminal epithelial cell-specific genes (Figure 2A).

To investigate cell type–specific histone methylation profiles, we employed a spatial clustering approach for the identification of ChIP-enriched regions using the SICER algorithm [28]. We identified 7,336 and 19,358 significantly K27-enriched islands in CD44+ and CD24+ cells from sample 1, respectively, using default conditions and FDR<0.001 as cutoff (details in Protocol S1). To examine the histone modification profiles of RefSeq genes, we analyzed the promoter regions of genes for overlap with K27- or K4-enriched islands. Using this approach we identified 1,182 K27-enriched genes in CD44+ cells, 716 (60.6%) of which was also K27-enriched in CD24+ cells, whereas 466 genes lost and 1,502 genes gained K27 mark during luminal lineage commitment (Figure 2B and Table S1).

Genes enriched for K27 mark in both or in each of the two cells types were functionally distinct based on DAVID bioinformatics (Figure 2C) and MetaCore [29] (Table S2). Several of the highest ranked pathways and processes unique for genes enriched for K27 only in CD24+ cells (CD24+/K27+) are related to stem cell function such as cyclic AMP, WNT, and TGFb signaling. These results indicate that K27 modifications regulate key signaling pathways in the two cell types relevant to progenitor and luminal epithelial cell functions.

To investigate whether genomic regions enriched for K27 or K4 marks only in CD24+ or CD44+ cells may contain binding sites for cell type–specific transcriptional regulators, we performed motif-search using Cistrome Analysis Pipeline Module (http://cistrome.dfci.harvard.edu/). We did not find any motifs significantly enriched in K4-enriched regions and found only a few motifs enriched in K27-marked regions (for example, TEAD1, SOX4, TCF3, and ZEB1 binding motifs are enriched in CD24+ specific K27 enriched regions) but the significance (z-score and p-value) for these motifs was low and none of them appeared to be cell type–specific. This result is not surprising, since ChIP-Seq data for multiple important transcriptional regulators (e.g., FoxA1) have demonstrated enrichment of binding sites in enhancer regions marked by histone H3K4me2 marks [30].

### Associations between gene expression and histone modification patterns

**Figure 3:**
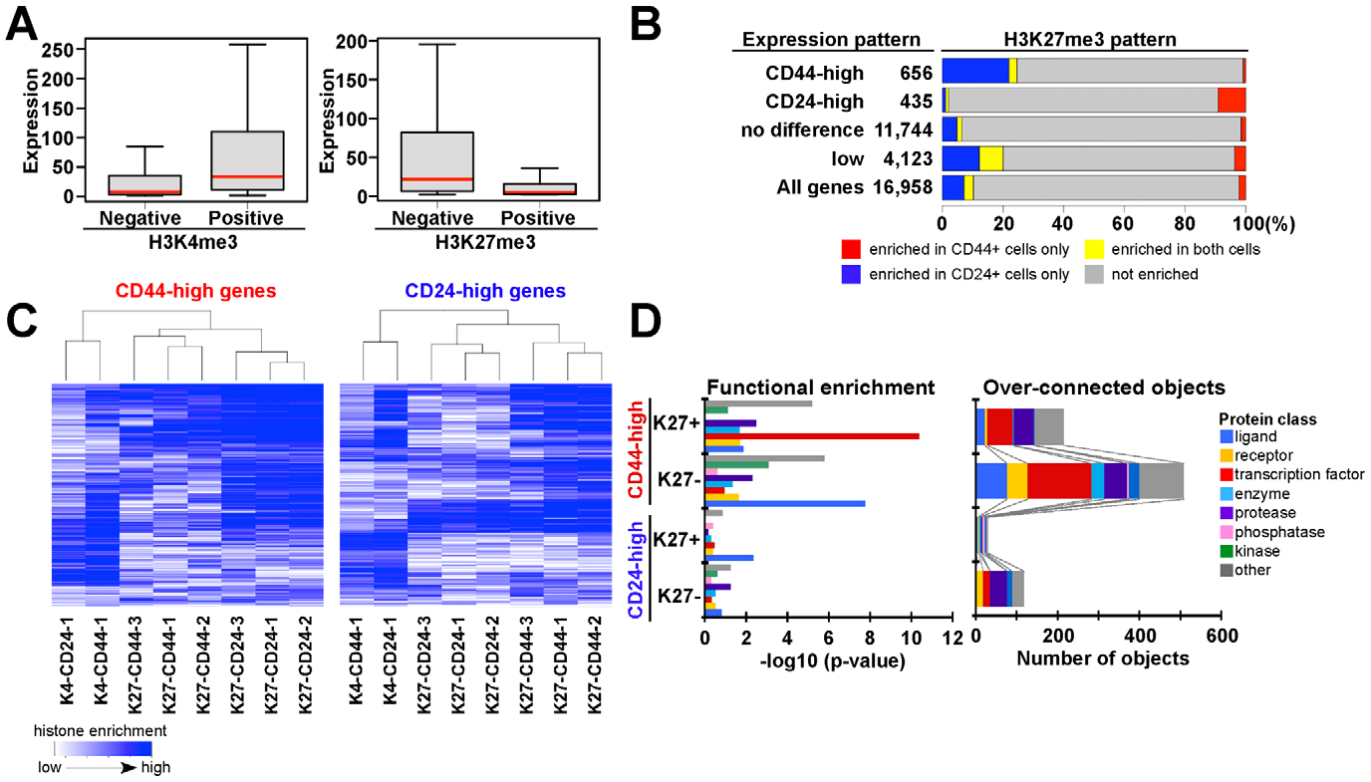
Associations between chromatin and cell type–specific expression patterns. (A) Overall correlation between histone methylation (K4, K27) and gene expression in CD44+ progenitors from sample 1. Box plot shows distribution of gene expression of corresponding genes in each category. Red bar: median, box: interquartile ranges and whisker; most extreme value within 1.5 times of box length. (B) Bar chart shows the correlation between cell type–specific K27 enrichment and gene expression patterns. Blue and red: K27-enriched only in CD24+ and CD44+ cells, respectively, yellow and gray: K27 enriched in both and neither cell type. (C) Differences in K27 patterns between CD24+ and CD44+ cells are consistent in three individual samples and distinct from that of K4 profiles. Heatmap depicting unsupervised clustering of histone modification patterns of genes highly expressed in CD44+ (656 genes) and CD24+ (435 genes) cells. Blue color indicates the level of enrichment for the indicated histone modification based on the ranking of ChIP-Seq read counts for each gene in each sample. (D) Differentially expressed genes that are enriched (K27+) or not enriched (K27-) for K27 were analyzed for relative enrichment with the indicated protein classes (left panel) and for relative connectivity (right panel). X-axes indicate –log10 p-values for enrichment with the listed protein classes (left panel) and the number of overconnected objects, defined as proteins with higher than expected number of interactions, in each functional category within each group (right panel), respectively.

Next, we investigated the associations between histone methylation and gene expression patterns by combining ChIP-Seq and SAGE-Seq data. Overall, histone methylation states and gene expression patterns demonstrated very good correlation as K4 and K27-enriched genes showed high and low expression levels, respectively, in both cell types (Figure 3A and Figure S3A, S3B).

To assess what fraction of genes was regulated by K27 modification in CD44+ and CD24+ cells, we evaluated K27 states for each gene in relation to its expression pattern. We first identified genes that were consistently differentially expressed between CD44+ and CD24+ cells (Figure S3C) and then categorized these into four groups: CD44-high, CD24-high, no difference, and not expressed. CD44-high genes showed K27 enrichment in CD24+ cells, whereas the reverse pattern was observed for CD24-high genes (Figure 3B and Table S3). These data suggest that 10–20% of the genes differentially expressed between CD44+ and CD24+ cells may be regulated by K27 modification. Besides some interindividual variability, the observed K27 histone methylation patterns were fairly consistent in cells isolated from three different individuals implying true cell type–specific differences and physiologic relevance (Figure 3C).

To investigate the function of the genes differentially expressed between CD24+ and CD44+ cells and differentially enriched for K27 (Table S4), we analyzed gene ontology and pathway annotation using MetaCore [29]. Interactome analysis assessing the relative connectivity of each protein in a dataset of interest with all human proteins (within the dataset or not) revealed that higher number of transcription factors, ligands, and proteases were overconnected (i.e., have significantly more one-step protein-protein interactions than expected) with CD44-high compared to CD24-high genes. Because relative connectivity is a measure of functional relevance of a protein for a dataset of interest, these data imply that CD44+ progenitor cell-specific genes might be regulated by more complex interactions than CD24+ luminal lineage-specific ones (Figure 3D). Furthermore, based on functional enrichment analysis by protein class, transcription factors were enriched only among CD44-high/K27+ genes (Figure 3D), suggesting that K27 modification may regulate key transcription factors important for mammary epithelial progenitor and luminal lineage-specific pathways.

### Genome-scale organization of H3K27me3-enriched regions

**Figure 4:**
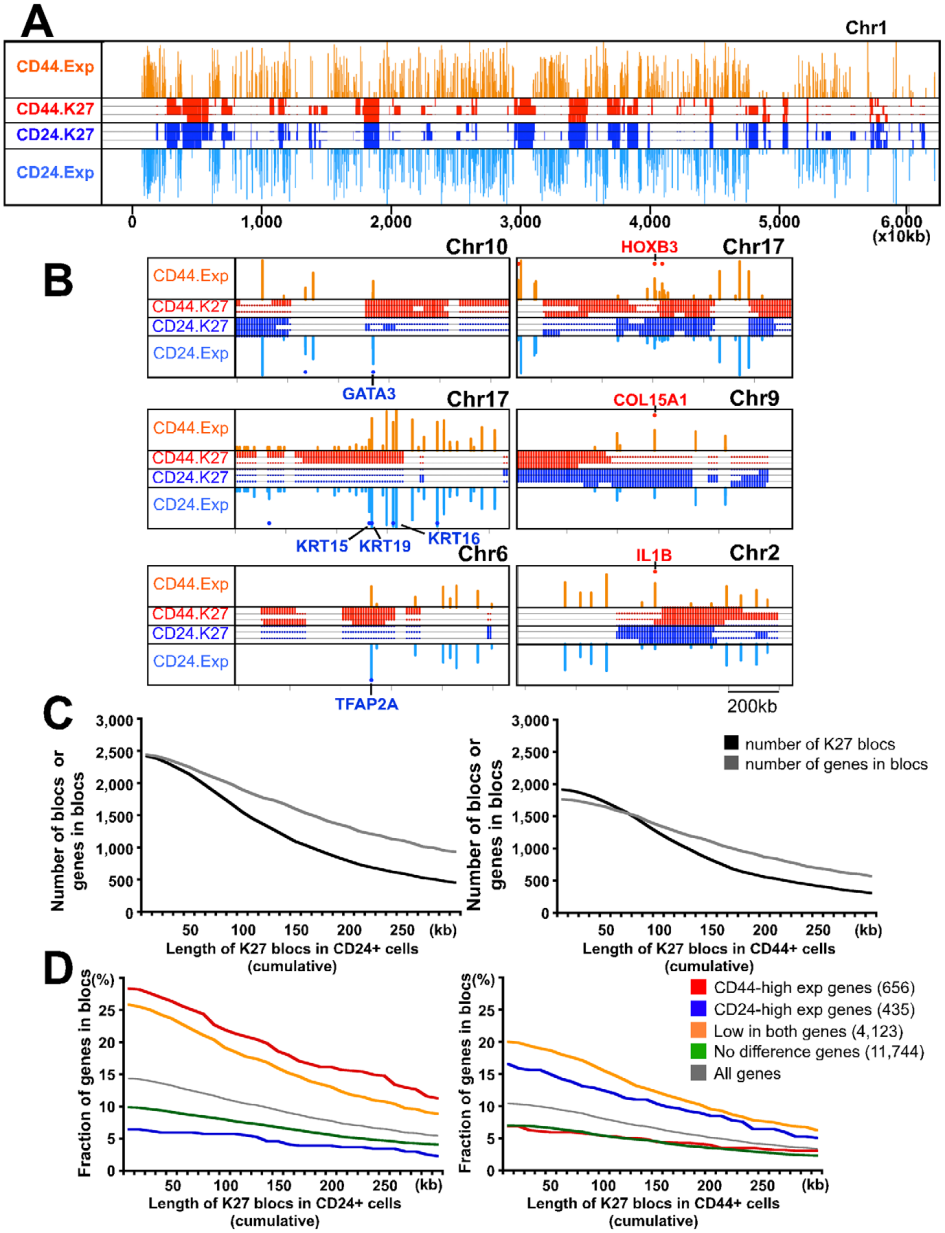
Genome-wide H3K27me3 patterns of human mammary epithelial progenitor and differentiated luminal cells. (A) Patterns of K27 enrichment and gene expression are mutually exclusive. Representative example of K27 distribution and gene expression in chr1. Data was analyzed using SICER algorithm [28] using 10 kb as window size. Significantly enriched regions and gene expression levels are plotted as colored lines across chromosome position. Red and blue lines represent K27 enrichment in CD44+ and CD24+ cell libraries from three different individuals, respectively. Gene expression levels are plotted across chromosome position. The height of line indicates the level of expression of corresponding gene. Orange and light blue: median expression in CD44+ and CD24+ samples, respectively. (B) Representative examples showing that distinct K27 distributions correlate with gene expression using the same plot as in panel A but at different scale. Red and blue dots indicate genes highly expressed (>2 fold difference) in CD44+ and CD24+ cells, respectively. Clear differences in K27 distribution between the two cell types are observed consistently in the regions where the selected genes are located. (C) Correlations between the number of K27 blocs and the number of genes in these blocs in CD44+ (left) and CD24+ (right) cells depending on setting K27 blocs at different sizes. X-axes indicate the threshold of length for defining K27 blocs, whereas y-axes show the number of K27 blocs and the number of genes within these blocs. (D) Fraction of genes with the indicated expression pattern located within K27 blocs in CD44+ and CD24+ cells.

During the course of our data analysis, we noted that K27-enriched regions tended to be broad rather than focal, and that this pattern was consistent within the same cell type and distinct between CD24+ and CD44+ cells. K27 distribution patterns were visualized by plotting enriched-only bins using 10 kb non-overlapping windows. Correlating with the clear differences of K27 patterns between CD44+ and CD24+ cells, genes located in these regions showed differential gene expression (Figure 4A, 4B). Gene density also differed significantly between K27-enriched and not-enriched regions as only a small fraction of genes was located in highly K27-enriched regions (Figure 4A). Thus, K27 enrichment and gene density (and expression levels) are mutually exclusive. The expression of genes within broad K27-enriched regions was fairly low and in some regions, a cluster of genes rather than a single gene had K27 mark implying a higher-level organization of the genome, chromatin, and gene expression patterns (Figure S4).

We further analyzed this and we identified 1,248 and 1,550 K27 blocs (broadly enriched regions) in CD44+ and CD24+ cells from sample 1, respectively, using 100 kb as threshold (Figure 4C). Many genes with important roles in stem cells and development (e.g., HOXB3, ZEB2, and RXRA) were located within blocs only in CD24+ cells (Figure 4B and Table S3), whereas 54 genes with known roles in luminal lineage differentiation (e.g., GATA3) showed the opposite pattern. Overall the number of CD44-high genes present in K27 blocs in CD24+ cells was higher than the number of CD24-high genes located in blocs in CD44+ cells (22.0 vs. 12.4%) (Figure 4D). Even genes with low/no expression in both cell types were more frequently located in K27 blocs in CD24+ than in CD44+ cells. This might indicate the coordinated silencing of progenitor cell-specific programs in CD24+ cells as the localization of genes in K27 blocs may ensure their synchronized regulation in a specific cell type and may also reduce their spurious activation due to transcriptional noise in other cells. The observed differences between the two cell types may suggest stricter transcriptional control in more differentiated cells.

### Chromatin patterns of distinct human cell types

**Figure 5:**
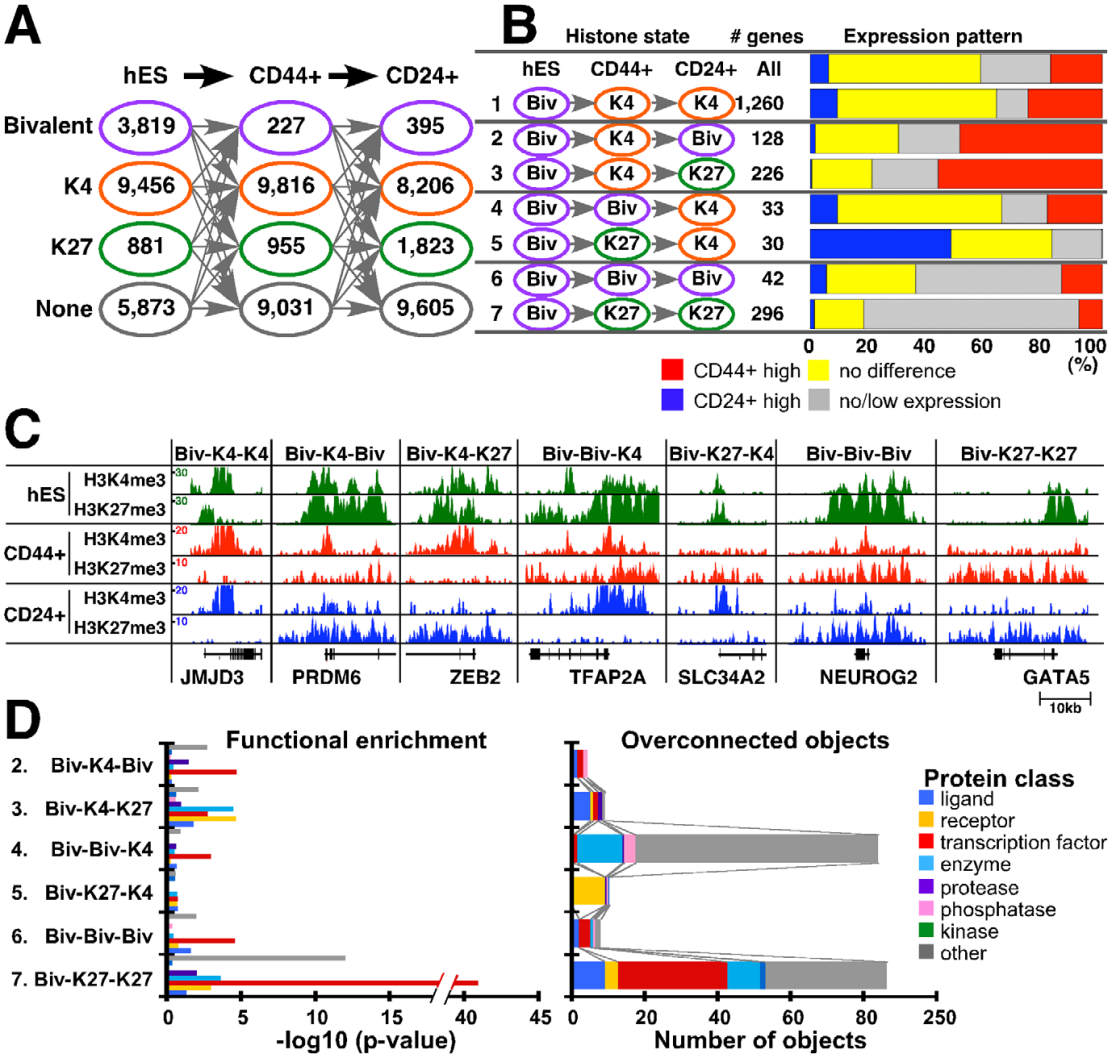
Changes in chromatin state and cell type–specific gene expression patterns. (A) Number of genes for each of the four possible chromatin states (i.e., bivalent – purple, K4 only – orange, K27 only – green, and neither – gray) in the indicated three cell types. (B) Potentially interesting differences in chromatin patterns. Bar chart shows associations between changes in chromatin-state and gene expression patterns. Each row indicates the type of chromatin-state change and the number of genes in each category. Blue and red: genes highly expressed in CD24+ and CD44+ cells, respectively (≥2-fold change). Yellow and gray: genes with ≤2-fold difference between CD24+ and CD44+ cells and with low/no detectable expression in either cell type, respectively. (C) Representative examples of genes in each category. ChIP-Seq tag counts for K4 and K27 modification in hES, CD44+, and CD24+ cells are shown. Total aligned tag count was scaled to 10 million and tag counts were averaged over a 10 bp window. (D) Functional enrichment analysis of genes within each chromatin pattern category (left panel) and the number of overconnected objects in each functional category within each group (right panel). X-axis indicates –log10 p-values for enrichment with the indicated protein class (left panel) and the number of overconnected objects (right panel), respectively. Definitions are the same as described in Figure 3D.

Several studies have shown that bivalent chromatin marks, defined as genes enriched both for K27 and K4, are important for the regulation of developmental genes in hESCs [3], [4]. More recent papers also reported the presence of these domains in other cell types including multiple different types of adult somatic [31] and cancer cells [32]. To investigate putative bivalent domains in human mammary epithelial cells, we analyzed our histone modification profiles and compared them with previously reported hESC data [3], [4], [20]. We classified all genes into four groups (bivalent, K4-only, K27-only, and neither) based on their enrichment for K4 and K27 marks (Figure 5A and Table S5). Using this classification hESCs had many (3,819) bivalent genes whereas the number of putative bivalent states was more limited in human mammary epithelial cells (Figure 5A). The number of genes showing neither or K4-only chromatin marks remained fairly constant; in contrast, the number of K27-only genes increased with increasing specification.

We analyzed changes in chromatin states and their correlation with expression patterns in the three cell types to define their potential biological relevance. There are 64 possible combinations of the four chromatin states in the three cell types (hES, CD44+, and CD24+ cells) and each pattern may have its own functional relevance. Thus, we examined each pattern individually and several of them appeared to be biologically interesting (Figure 5B and Figure S5A). For example, 1,260 genes showed bivalent-K4-K4 (chromatin states in hES, CD44+, and CD24+ cells) pattern (category 1) despite differences in expression between CD24+ and CD44+ cells. 354 genes associated with bivalent mark in hESCs lost K27 mark in CD44+ and regained it in CD24+ cells (categories 2 and 3). Corresponding to this K27 pattern, half of genes in these categories appear to be activated and showed higher expression in CD44+ cells. However, 128 genes (category 2) became bivalent state again, whereas 226 genes lost K4 and became K27-only in CD24+ cells (category 3). Similarly, genes in category 4 and 5 lost K27 and became active K4 state in CD24+ cells, and 30–40% of them showed higher expression in CD24+ compared to CD44+ cells. However, 30 genes lost K4 mark and became K27-only in CD44+ cells (category 5), whereas 33 genes kept K4 mark and remained bivalent in CD44+ cells (category 4). These data suggest that a subset of genes might preferentially keep K4 mark regardless of repression by K27. In the same context, among genes that were bivalent in hESCs, 42 retained bivalent state (category 6) and 296 became K27-only (category 7) in both CD44+ and CD24+ cells, but they still showed some expression in mammary epithelial (particularly in CD44+) cells (Figure S5A). Examples for histone modification patterns of selected genes in each category are depicted in Figure 5C.

We were not able to obtain experimental evidence (e.g., sequential ChIP) to prove that the putative bivalent domains we identified are truly bivalent. Thus, to gain additional support we also examined chromatin states for these genes in other human cell types such as NHEK epidermal keratinocytes and HUVEC umbilical vein endothelial cells based on public ChIP-Seq data. Genes that showed bivalent or K27-only (categories 6 and 7) patterns both in CD44+ and CD24+ cells tended to have the same pattern in other cell types as well (Figure S5B). These data suggest that a subset of genes keep bivalent mark in differentiated cells.

To investigate the functional relevance bivalent genes, we compared genes in categories 2–6 using Metacore [29]. Overall, transcription factors were enriched in bivalent domains in all cell types and many of the ones active in ESCs become K27-only associated during tissue-specific differentiation (Figure 5D and Table S6). Interactome analysis revealed a number of developmental transcription factors overconnected (i.e., having more interactions than expected) with genes from Biv-K4-Biv and Biv-K4-K27 sets. In the Biv-Biv-K4 vs. Biv-K27-K4 comparison we also observed a similarly interesting development bias as the Biv-Biv-K4 set was regulated by two homeobox transcription factors (HOXA2 and PITX2), whereas Biv-K27-K4 had no overconnected genes. Moreover, ontology enrichment showed that Biv-K27-K4 is enriched with developmental processes, TGFß and ERBB family signaling pathways, consistent with the presumed importance of these in mammary epithelial cells (Table S6). Finally the Biv-K27-K27 set is overconnected with a large number of developmentally important transcription factors including OCT4 and SOX2 two transcription factors critical for maintaining pluripotent state in ESCs [33].

### Cell type–specific differences in DNA methylation patterns

To explore cell type–specific differences in DNA methylation patterns and their relationship to histone modification and gene expression, we analyzed the DNA methylation profiles of CD44+ and CD24+ cells using MSDK-Seq utilizing the BssHII methylation sensitive restriction enzyme. By analyzing 32,453 observed MSDK sites, 48.5% of which were located within +/−5 kb from RefSeq TSS, we identified a total of 1,256 DMRs (differentially methylated BssHII restriction enzymes sites) that displayed significant (p<0.01, Poisson margin model) differences between CD44+ and CD24+ cells (405 and 851 were hyper-methylated in CD24+ and CD44+ cells, respectively). To explore the variability of DMRs in CD24+ and CD44+ cells across multiple individuals, we performed hierarchical clustering of the read counts for each of the 1,256 significant DMRs across all samples. The heatmap of this clustering shows that most of the variation is between CD24+ and CD44+ cells and the degree of variation across multiple individuals is less pronounced (Figure S6A). We also experimentally validated several candidate regions by qMSP (quantitative methylation-specific PCR) in multiple independent samples (Figure S6B).

**Figure 6:**
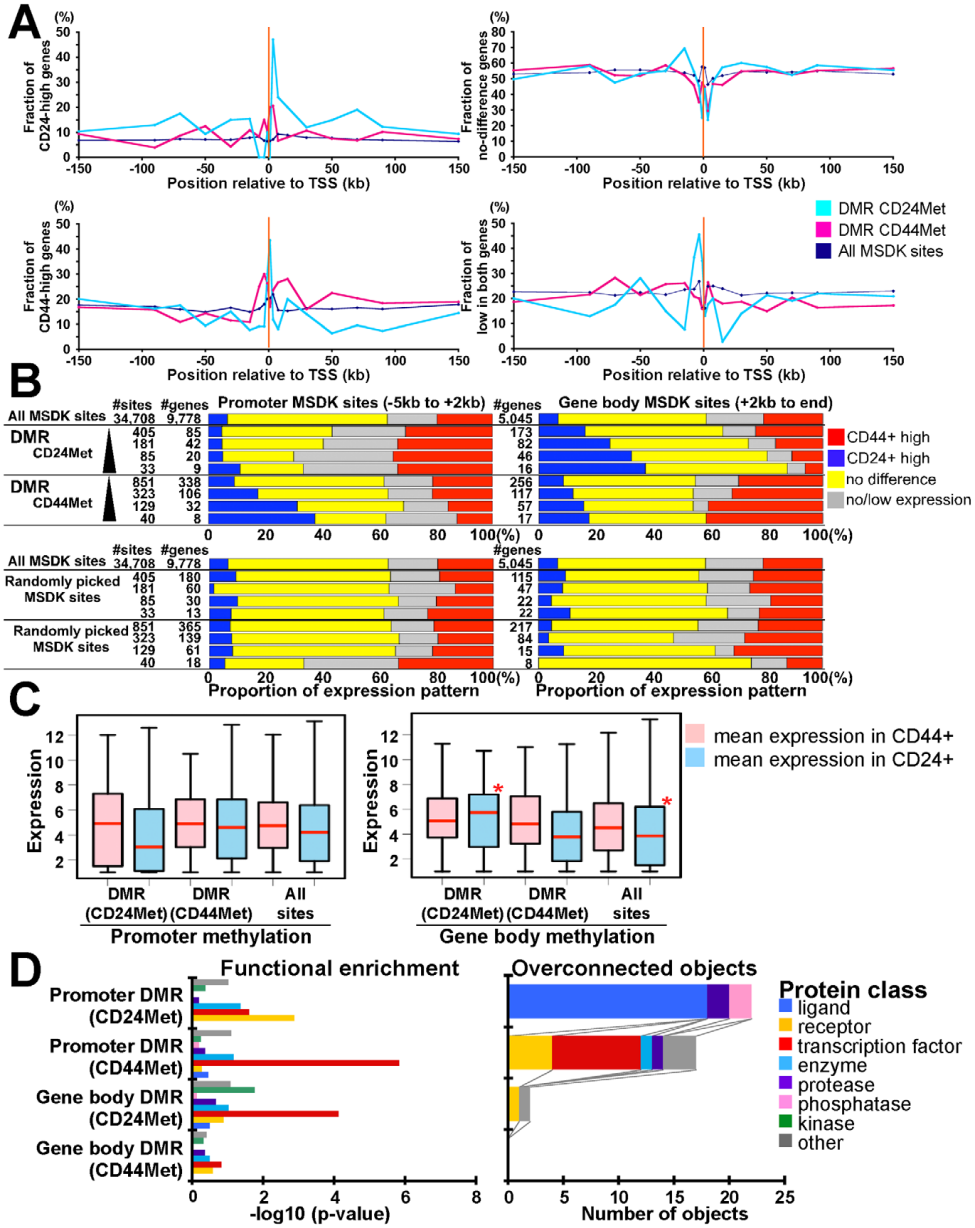
Combined view of DNA methylation and gene expression patterns. (A) Associations between fraction of genes highly expressed in CD24+ or CD44+ cells and the location of DMRs (p<10^−5^) in the two cell types relative to TSS. Y-axes show fraction of genes in the four different gene expression groups (i.e. CD24-high, CD44-high, no difference, and not expressed) relative to the location of DMRs hypermethylated in CD24+ (blue line) or CD44+ (red line) cells and all MSDK sites used as control. (B) Differentially (≥2-fold difference) expressed genes are enriched in DMRs. Bar charts show associations between genes with indicated DMR and gene expression patterns as described in panel A. Genes in each gene set have DMR (indicated left side) within promoter region (−5 kb from TSS to +2 kb, left panel) or in gene body (+2 kb to end, right panel). The number of MSDK sites and associated genes in each group is indicated. We used four different cut-offs for DMRs, 2, 5,10, 20 (-log10 p-value) from top to bottom (black triangle). Randomly picked MSDK sites did not show any enrichment pattern. (C) Correlation between mean gene expression levels in relation to promoter and gene body methylation in CD24+ and CD44+ cells. Red stars mark statistically significant differences relative to all MSDK sites. (D) Functional enrichment analysis (left panel) of genes associated with promoter and gene body DMRs in CD24+ and CD44+ cells and the number of overconnected objects in each functional category within each group (right panel). X-axis indicates –log10 p-values for enrichment with the indicated protein class (left panel) and the number of objects (right panel), respectively. Definitions are the same as described in Figure 3D.

To examine whether DMRs are enriched for differentially expressed genes, we analyzed associations between the presence of DMR at various positions (−150 to +150 kb) relative to TSSs (transcriptional start sites) and gene expression patterns (Figure 6A). Genes were divided into four groups (CD44+−high, CD24-high, not differentially expressed, and not expressed in either cell type) based on their expression levels and into three groups (DMR CD24Met - hypermethylated in CD24+ relative to CD44+ cells, DMR CD44Met - hypermethylated in CD44+ relative to CD24+ cells, and All MSDK sites) based on their DNA methylation patterns. We found that in general DMRs immediately upstream and near promoters (−5 kb to +2 kb from TSS) showed negative association between the level of methylation and gene expression indicating a repressive effect. In contrast, DMRs in gene body (defined as +2 kb from TSS to end of gene) showed positive association between methylation and expression suggesting that methylation in gene body is associated with increased gene expression in agreement with previous data [34], [35]. These associations were found in both CD44+ and CD24+ cells (Figure 6A). However, the fraction of CD24-high and CD44-high genes varied greatly with respect to the presence of DMRs in the two cell types at different positions relative to TSS. Thus, we classified DMRs based on their relative position to TSS and identified 106 and 117 genes with promoter and gene body methylation in CD44+ cells, respectively, and 42 and 82 genes in CD24+ cells using p<0.00001 as a threshold for DMRs (Table S7). By analyzing the cell type–specific expression of these genes we found several potentially interesting differences between CD24+ and CD44+ cells. First, the effect of gene body DNA methylation is more pronounced in CD24+ cells as the expression of a higher fraction of CD24+ cell-specific genes is influenced by DNA methylation in CD24+ cells than the expression of CD44+ cell-specific genes in CD44+ cells (Figure 6A–6C). Randomly selected MSDK sites did not show any association with cell type–specific gene expression in either cell type (Figure 6B). Second, even promoter methylation appears to have a stronger silencing effect on gene expression in CD24+ compared to CD44+ cells as the mean expression of genes associated with promoter DMRs in CD24+ cells is lower in CD24+ cells (Figure 6C). These results imply a cell type–specific difference in DNA methylation and in the regulation of genes that differentiate the two cell types. However, further more comprehensive studies are needed in order to determine whether DNA methylation has different effects on gene expression in CD24+ and CD44+ cells, as MSDK-Seq only sampled a fraction of the genome. Ideally DNA methylation patterns would have to be analyzed at single nucleotide resolution in both cell types as MSDK-Seq only sampled a fraction of the genome: out of total 28,226 CpG islands we analyzed 13,715 (48.6%).

Ontology analysis of genes associated with DMRs in CD24+ and CD44+ cells showed significant (p<0.05, Fisher's exact test) differences between genes with gene body and promoter methylation in both cell types (Figure 6D). Interestingly, genes with promoter hypermethylation in CD44+ and genes with gene body hypermethylation in CD24+ cells were enriched for transcription factors implying that the expression of transcription factors relevant in CD24+ cells (e.g., GATA3) are suppressed by promoter methylation in CD44+ cells and are positively regulated by gene body methylation in CD24+ cells. No similar observations were found for CD44+ cell-related transcription factors potentially reflecting differences in the relative importance of DNA methylation for establishing cell type–specific gene expression.

### Integrated molecular view of mammary epithelial cells

**Figure 7:**
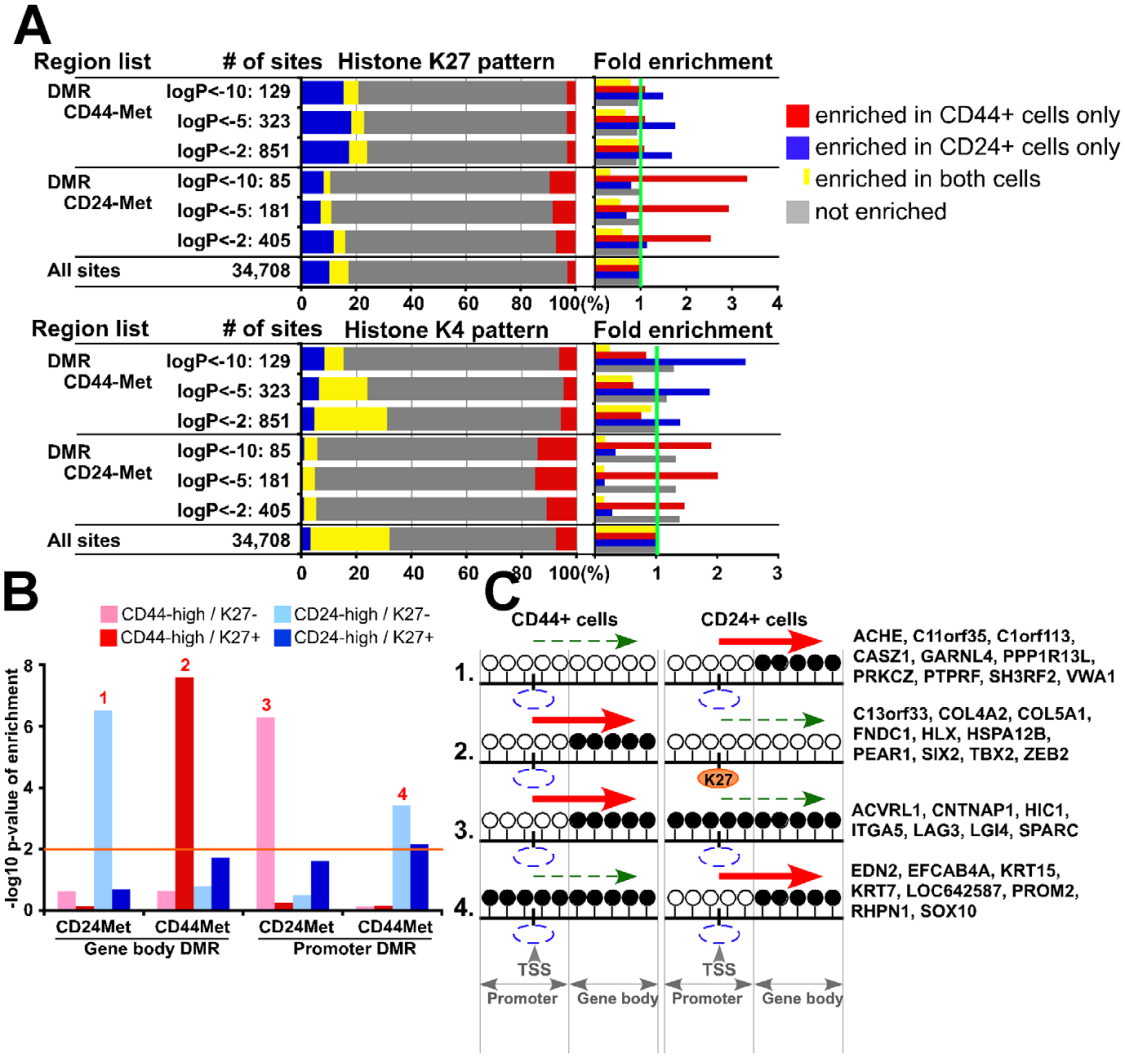
Integrated view of genome-wide gene expression and DNA and histone methylation patterns. (A) Genomic regions (+/− 5 kb and +/− 0 kb from DMR for K27 and K4, respectively) associated with DMRs in one cell type (e.g., CD44+ cells) are enriched for K4 or K27 mark in the other (e.g., CD24+ cells). Bar chart shows observed/expected ratio of the indicated MSDK sites with the designated K27 and K4 patterns between CD44+ and CD24+ cells. (B) Associations between gene expression and histone and DNA methylation patterns. Y-axis shows the –log10 p-value of enrichment for genes with the indicated expression and histone modification pattern in gene body and promoter DMRs. Orange line indicates –log10 (p value) of statistical significance, numbers 1–4 mark significantly enriched patterns. (C) Schematic models depicting possible changes in DNA methylation and K27 enrichment during CD44+ to CD24+ cell differentiation and their effect on gene expression based on data presented in panel B. Examples of genes within each group are listed. White (unmethylated) and black (methylated) circles indicate potential DNA methylation sites (i.e., CpG) in the promoter and gene body, blue and orange ovals represent lack and presence of K27 mark, respectively. Red and dashed green arrows indicate increased and decreased gene expression, respectively.

To integrate all three types of genomic data, we evaluated DMRs and differentially expressed gene sets in relation to the presence of K27 marks and found several specific combinations of patterns that were significantly (p<0.05, Fisher's exact test) enriched (Table S7). First, we investigated associations between DMRs and nearby (−/+ 5 kb from DMR) K27 marks and found that DMRs hypermethylated in CD24+ cells were enriched for K27 mark in CD44+ but not in CD24+ cells (Figure 7A). The same pattern was observed for K4 marks around DMRs in both cell types: regions hypermethylated in CD24+ cells were enriched for K4 in CD44+ cells and vice versa.

Next, we also included gene expression data in our analysis and due to the differential effect of DMR depending on its location with respect to TSS (gene body or promoter), we analyzed these regions separately. For promoter region associated DMRs in both cell types we found that hypermethylated DMRs were enriched for genes highly expressed in the other cell type (e.g., promoter hypermethylated DMRs in CD44+ cells were enriched for genes highly expressed in CD24+ cells) and not enriched for K27 mark (Figure 7B and Table S7). For gene body methylation, we detected a more complex pattern. DMRs hypermethylated in CD44+ cells were enriched for CD44-high genes with K27 mark, whereas DMRs hypermethylated in CD24+ cells were enriched for CD24-high genes without K27 mark. Hypothetical models explaining the different modes of regulation and examples of genes for each significantly enriched integrative pattern are depicted in Figure 7C. These results suggest that the combined effects of DNA and K27 methylation are different in different cell types and depend on the location of DMR relative to TSS.

## Discussion

We have generated the first comprehensive epigenomic profile of human mammary epithelial cells by analyzing gene expression, DNA and histone methylation patterns of CD44+ progenitor and CD24+ luminal lineage-enriched cells. Our data revealed several interesting cell type–specific differences between CD44+ and CD24+ cells and identified key candidate regulators of mammary epithelial cells. The main findings of our study are as follows: histone K27 and DNA methylation patterns are distinct between CD44+ and CD24+ cells and their relative importance in regulating cell type–specific gene expression may also be different. Genes mutually exclusively repressed by K27 mark in CD44+ and CD24+ cells are frequently localized in K27 blocs, relatively gene-poor large genomic regions enriched in K27 mark, that may ensure the coordinated cell type–specific regulation of these genes. Putative bivalent chromatin domains are present in all cell types and genes marked by them are enriched for transcription factors with key roles in development. Gene body and promoter DNA methylation targets different sets of genes and may have different effects on their expression in CD44+ and CD24+ cells.

### Regulation of key transcription factors and pathways by K27 in mammary epithelial cells

Genome-wide analysis of chromatin states in defined cell populations provides comprehensive information about developmental potential and differentiation states especially when combined with global gene expression patterns. However, due to the requirement of large numbers of cells for ChIP, thus far most of these studies have been performed using cultured cells that may show non-physiologic histone modification patterns. We employed our newly developed method to gene expression, DNA and histone methylation profiling of cells isolated from human breast tissue overcoming these technical limitations and accelerating progress in this area.

Our ChIP-Seq results identified many transcription factors regulated by K27 that might play key roles in mammary epithelial lineage commitment and differentiation. Genes marked with K27 in CD24+ luminal but not in CD44+ progenitor-enriched cells include numerous HOX genes, GLI1, HES3, HES7, HEYL, and TCF4, all known regulators of stem cells, as well as several EMT (epithelial to mesenchymal transition) inducing transcription factors such as GSC, SNAI2, TWIST1, and ZEB2. This latter finding correlates with studies describing the induction of CD44+ stem cell-like cells both in normal and neoplastic breast epithelial cells by the exogenous overexpression of these genes [14], [36]. In addition to transcription factors, components of cAMP, TGFß, FGF, PIP3K, and integrin signaling pathways implicated in embryonic and hematopoietic stem cells, were silenced by K27 in CD24+ cells suggesting their potential importance in mammary epithelial progenitors. In contrast, genes highly expressed in CD24+ and marked by K27 in CD44+ cells included transcription factors GATA3 and TFAP2A. GATA3 is a key transcriptional regulator of luminal epithelial cell fate [26], [27], wheras the role of TFAP2A in mammary epithelial cells have not been analyzed. Thus, the functional analysis of the genes we identified will further our understanding of the regulation of human mammary epithelial progenitors and their differentiation.

### Changes in bivalent domains during mammary epithelial and luminal lineage commitment

Bivalent domains were defined as genomic regions enriched for both active K4 and repressive K27 histone marks thought to keep genes silent but poised for activation in ESCs [3]. The persistence of bivalent domains was demonstrated in ASCs and even in terminally differentiated cells [20], [37]. However, all these studies used culture-based differentiation systems and did not analyze cells isolated from primary human tissue samples.

To investigate changes in bivalent domains in mammary epithelial cells we analyzed genes associated with bivalent domains in hESCs and in our datasets. Compared to hESCs, the number of genes targeted by bivalent domains was substantially lower in CD44+ and CD24+ cells, whereas the fraction of K27-only genes increased with decreasing developmental potential and increasing specification (Figure 5A). By analyzing differences in chromatin patterns in ESC, CD44+, and CD24+ cells and the function of the genes targeted by these, we made several interesting observations.

Among genes that are differentially enriched for K27 between CD24+ and CD44+ cells a subset keeps K4 mark and remain bivalent (Biv-K4-Biv) in CD24+ cells others tend to lose K4 and become K27-only (Biv-K4-K27). Genes in both of these categories are activated and expressed in CD44+ cells. We found that transcription factors were more enriched in the Biv-K4-Biv group and these included many homeobox (e.g., HOXC9 and HOXD1) and other genes with known developmental function (e.g., GSC and HES7). We hypothesize that these genes might be temporally activated in CD44+ cells by the removal of K27 mark but then go back to bivalent state in CD24+ cells potentially because they might have to be activated in various situations more frequently than genes in the Biv-K4-K27 group. Comparison of the Biv-Biv-K4 and Biv-K27-K4 gene sets gave essentially the same results: transcription factors (e.g., FOXC1, TFAP2A, TFAP2C) were enriched only in the Biv-Biv-K4 group. The removal of K27 mark via the coordinated action of histone demethylases and methylases at a defined time point during mammary epithelial differentiation is likely to be important for the regulation of these transcription factors.

We attempted to experimentally validate these putative bivalent domains by sequential ChIP, but were unsuccessful likely due to limiting cell numbers. However, based on public ChIPSeq data, genes that showed bivalent patterns both in CD44+ and CD24+ cells tended to have the same pattern in other cell types as well (Figure S5B). These data suggest that the bivalent marks we observed are likely to be true bivalent domains, but the possibility of the coexistence of two cell populations showing either K4 or K27 mark [38] cannot be excluded.

### Differences in K27 enrichment and DNA methylation relative to cell type–specific gene expression

We show that the expression of different sets of genes is regulated by DNA methylation and K27 in CD44+ and CD24+ cells and that gene body and promoter methylation have differing effects on gene expression in the two cell types. Furthermore, the association between DNA methylation and gene expression is more pronounced in CD24+ cells. Specifically, the expression of a large fraction of CD24-high genes is negatively and positively associated with promoter and gene body methylation, respectively, whereas similar observations were found for a much smaller portion of CD44-high genes (Figure 6A–6C). We also found that genes highly expressed in CD44+ cells are preferentially enriched for K27 marks in CD24+ cells (Figure 3B and Figure 4D). Even genes that show low or no expression in both cell types are preferentially associated with promoter methylation in CD24+ compared to CD44+ cells (Figure 6A). Because DNA methylation is a more stable epigenetic modification than chromatin patterns, these data suggest that the cellular state of CD24+ luminal epithelial cells is more stable than that of CD44+ progenitor-enriched cells.

The interplay between K27 and DNA methylation also showed some interesting potential cell type–specific differences. For example, in CD24+ cells DNA methylation in gene body is associated with the higher expression of CD24-high genes without K27 mark (models 1 in Figure 7B, 7C), whereas in CD44+ cells this was true only for CD44-high K27-enriched genes (model 2 in Figure 7B, 7C). This latter model (model 2 in Figure 7C) suggests a putative mechanism whereby losing gene body methylation may lead to loss of gene expression in CD24+ cells and this is associated with the gain of K27 mark in the promoter region. PRC2 and DNMTs might collaborate in the repression of this group of genes that includes ZEB2, SIX2, HLX, and TBX2 transcription factors. On the other hand, the repressive effect of promoter methylation affected gene expression without K27 mark in the both cell types (models 3 and 4 in Figure 7C), suggesting that K27 might have nothing to do with this type of repressive mechanism.

In summary, our integrated global view of chromatin states, DNA methylation, and gene expression patterns of human mammary epithelial and luminal lineage commitment provides a framework for the identification and functional characterization of genes with key roles in these processes. However, there are also two main limitations of our study. First, our DNA methylation data is limited to a fraction of the genome, since based on MSDK-Seq we only evaluated the methylation status of the recognition site of the BssHII enzyme. Second, the cell fractions used for the study are not homogenously pure. Whole genome sequencing of bisulfite-treated genomic DNA isolated from single cells would overcome these limitations and with the fast pace of technical advances these types of studies may be possible in the near future.

## Materials and Methods

### Ethics statement

Fresh normal breast tissue specimens were collected at Harvard-affiliated hospitals (Boston, MA) and Johns Hopkins University (Baltimore, MD). All human tissue was collected using protocols approved by the Institutional Review Boards.

### Tissue samples and primary culture

Fresh tissue samples were immediately processed for immunomagnetic purification, and RNA and DNA was prepared essentially as previously described [16], [17].

### SAGE-Seq, MSDK-Seq, and ChIP-Seq sample preparation and data analysis

Details of SAGE-Seq, MSDK-Seq, and ChIP-Seq procedures and data analysis are posted on our lab's website (http://research4.dfci.harvard.edu/polyaklab/protocols_linkpage.php) or included in Protocol S1. All data was deposited to GEO under accession # GSE26141 and processed data is available at our lab website: http://research4.dfci.harvard.edu/polyaklab/links.php.

## Supporting Information

Figure S1 Comparison of small-scale and standard ChIP-Seq protocols. (A) Schematic outline of ChIP-Seq protocol. Critical steps that required optimization (red), DNA purification steps (blue), and quality control qPCR steps (green) are indicated. (B) Correlation of small-scale ChIP-Seq data between the same cell type from two different individual (left) and the same breast tumor purified two different ways (right).
(1.43 MB TIF)

Figure S2 Confirmation of cell purity and histone methylation patterns. (A) We performed qRT-PCR for known markers of CD44+ progenitor (red) and CD24+ differentiated luminal epithelial (blue) cells to confirm the success of the purification procedure. Part (15%) of the fractionated cells was used for RNA preparation whereas the remaining fraction (85%) was used for the generation of ChIP-Seq libraries. (B) The quality of ChIPed DNA is evaluated by qPCR during ChIP-Seq library preparation before and after PCR amplification and after size selection steps. Bar plots depict representative results for K27 in CD24+ (top panel) and CD44+ (bottom panel) cells. Y-axis indicates enrichment relative to negative control (RPL19 promoter region); KCNA1, HOXA13, and LHX1 are putative K27-enriched genes, whereas CDH1 and VIM are CD24+ and CD44+ cell type–specific genes, respectively.
(0.31 MB TIF)

Figure S3 Correlation between histone modification and gene expression patterns in each sample. (A) Scatter plots comparing the level of histone modification and the level of gene expression. Each dot represents a gene. X-axis indicates mapped read counts around promoter region for the indicated histone mark and Y-axis indicates the median expression of the corresponding gene in the indicated libraries. (B) H3K27me3 and H3K4me3 states around gene promoter regions negatively and positively correlate with corresponding gene expression levels, respectively. (C) SAGE-Seq analysis of CD44+ progenitor-enriched and CD24+ luminal epithelial cells. Dendograms depicting the relatedness of the indicated samples. Hierarchical clustering was applied to SAGE-Seq data of selected cell type–specifically expressed genes. Heatmaps show consistently differentially expressed genes between CD24+ and CD44+ cells. We selected 435 genes consistently highly expressed in CD24+ cells and 656 genes consistently highly expressed in CD44+ cells.
(1.63 MB TIF)

Figure S4 Examples of genes contained in K27 blocs. Patterns of K27 enrichment and gene expression are mutually exclusive. Examples showing clusters of genes located in K27 blocs and potentially silenced by this modification. Data was analyzed using SICER algorithm [28] using 10kb as window size. Significantly enriched regions and gene expression levels are plotted as colored lines across chromosome position. Red and blue lines represent K27 enrichment in CD44+ and CD24+ cell libraries from three different individuals, respectively. Orange and light blue: median expression in CD44+ and CD24+ samples, respectively. The height of line indicates the level of expression of corresponding gene. Red and blue dots indicate genes highly expressed (>2-fold difference) in CD44+ and CD24+ cells, respectively.
(0.69 MB TIF)

Figure S5 Chromatin states for “Biv-Biv-Biv” and “Biv-K27-K27” genes in different cell types. (A) Mean expression of genes (right bar graph) with the indicated chromatin pattern (left panel) in CD24+ and CD44+ samples. Wilcoxon rank sum test was performed to identify significant differences between CD44+ and CD24+ samples within the same group and between-groups. Asterisks indicate significant differences. (B) Representative examples of ChIP-Seq tag distribution in different cell types analyzed by the ENCODE project for several genes where we observed either bivalent or K27 mark in CD44+ and CD24+ cells. Total aligned tag count in each ChIP-Seq library was scaled to 10 million and tag counts averaged over a 10 bp window are shown in the Y-axis. NEUROG2, LHX4, and TFAP2A tend to keep K4 mark, whereas GATA5 tends to loose K4 mark in various cell types.
(1.30 MB TIF)

Figure S6 Examples of MSDK tag counts for selected genes with DMRs and their experimental validation and associations between DMRs and gene expression. (A) Heatmap depicting the clustering of samples based on tag counts for the 1,256 DMRs we identified. (B) Left: Schematic view of the indicated genomic region based on UCSC genome browser. Location of CpG islands, BssHII and NlaIII recognition sites, positions of primers used for qMSP are indicated. Right: Experimental validation of the indicated DMRs by qMSP. Cells purified from four independent individuals (N37, N38, 77358, and N48) are analyzed. Bars indicate methylation in CD44+ and CD24+ cells relative to *ACTB*. (C) Enrichment of DMRs in differentially expressed gene sets depending of the location of DMR relative to TSS. Observed/Expected ratios are shown for each combination. Expression pattern of each gene set is shown as bar chart. Genes in each gene set have indicated DMR (left panel:CD24-hypermethylaed DMR, middle: CD44-hypermethylaed DMR, right: any MSDK sites) in indicated location relative to their TSSs. red: genes highly expressed genes in CD24+ and CD44+ cells, respectively (≥2-fold change). Yellow and gray: genes with ≤2-fold difference between CD24+ and CD44+ cells and with low/no detectable expression in either cell type, respectively.
(0.92 MB TIF)

Protocol S1 Description of human tissue samples used for the generation of SAGE-Seq, ChIP-Seq, and MSDK-Seq libraries and number of aligned reads in each ChIP-Seq library and number of total tags in SAGE-Seq and MSDK-Seq libraries.
(0.23 MB DOC)

Table S1 List of genes enriched for K27 mark in CD24+ or CD44+ cells or in both cell types. The excel file contains three worksheets (CD44+K27+, CD24+K27+, and K27+ in both). Gene symbol, RefSeq ID, approved name, and chromosomal location are indicated.
(0.40 MB XLS)

Table S2 Summary of GeneGo pathway, network, and interactome analysis for K27-enriched genes. The excel file contains multiple worksheets. GeneGo processes and canonical pathway maps are listed with p-values indicating the significance of enrichment for K27 enriched genes. Functional enrichment analysis by protein class. r: number of genes showing indicated class in the list, n: total number of genes in the list, R: number of genes showing indicated class in the background list, N: total number of genes in the background list, mean value for hypergeometric distribution (n*R/N), z-score: z-score ((r-mean)/sqrt(variance)), p-value probability to have the given value of r or higher (or lower for negative z-score). Average connectivity, number of overconnected objects, and name of overconnected objects for each list.
(1.30 MB XLS)

Table S3 List of differentially expressed genes enriched in K27me3 or K4me3 histone modifications. The file contains four worksheets. (1) CD44-high/K27+ genes: genes highly expressed in CD44+ cells and K27-enriched in either or in both cell types. (2) CD44-high/K27- genes: genes highly expressed in CD44+ cells and not K27-enriched in either cell type. (3) CD24-high/K27+ genes: genes highly expressed in CD24+ cells and K27-enriched in either or in both cell types. (4) CD44-high/K27- genes: genes highly expressed in CD24+ cells and not K27-enriched in either cell type. Gene symbol, RefSeq ID, approved name, chromosomal location, and location within K27 bloc (Y = yes and N = No) are indicated.
(0.19 MB XLS)

Table S4 Summary of GeneGo pathway, network, and interactome analysis for Table S3. The file contains multiple worksheets. Legend is the same as that of Table S2.
(0.54 MB XLS)

Table S5 List of genes affected by chromatin pattern changes. The file contains multiple worksheets. Genes showing specific chromatin pattern changes in hESC, CD44+ and CD24+ cells as described in Figure 5B. (A) Biv-K4-K4 (in hESC, CD44+ and CD24+, respectively), (B) Biv-K4-Biv, (C) Biv-K4-K27, (D) Biv-Biv-K4, (E) Biv-K27-K4, (F) Biv-Biv-Biv, (G) Biv-K27-K27. Gene symbol, RefSeq ID, gene description, and chromosomal location are indicated.
(0.30 MB XLS)

Table S6 Summary of GeneGo pathway, network, and interactome analysis for Table S5. The file contains multiple worksheets. Legend is the same as that of Table S2.
(0.68 MB XLS)

Table S7 List of genes associated with DMRs and K27-enriched regions. The file contains seven worksheets. Complete list of DMRs identified in CD44+ (1) and CD24+ (2) cells. List of DMR (-log10(p-value)>5) hypermethylated in CD24+ cells (CD24Met) (1) and DMR hypermethylated in CD44+ cells (CD44Met) (2). ID, -log10 (p-value), chromosomal location of the DMR, genes with the DMR in their promoter regions, genes with the DMR in their gene body regions, overlap with CpG island (P: yes, N: no), tag count in each MSDK-Seq library are indicated. Genes with DMR hypermethylated in CD24+ cells (CD24Met) in genebody (3) and promoter (4) and genes with DMR hypermethylated in CD44+ cells (CD44Met) in genebody (5) and promoter region (6). Gene symbol, RefSeq ID, gene description, and chromosomal location are indicated. (7) List of genes differentially expressed and associated with DMR and histone modifications. Genes showing DMR in promoter or genebody and also showing differentially expression between CD44+ and CD24+ cells. Gene symbol, RefSeq ID, gene description, chromosomal location, differential expression patterns and histone K27 enrichment (P: positive, N: negative) in their promoter regions in CD44+ cell and CD24+ cells are indicated. Data from Tables S3 and S5 are integrated into this table.
(0.23 MB XLS)
