# Supplementary figures and images for: Epigenetic Regulation of Cell Type–Specific Expression Patterns in the Human Mammary Epithelium
Source: PLoS Genet. 2011 Apr 21;7(4):e1001369. doi: 10.1371/journal.pgen.1001369 (PMC3080862; doi:10.1371/journal.pgen.1001369)

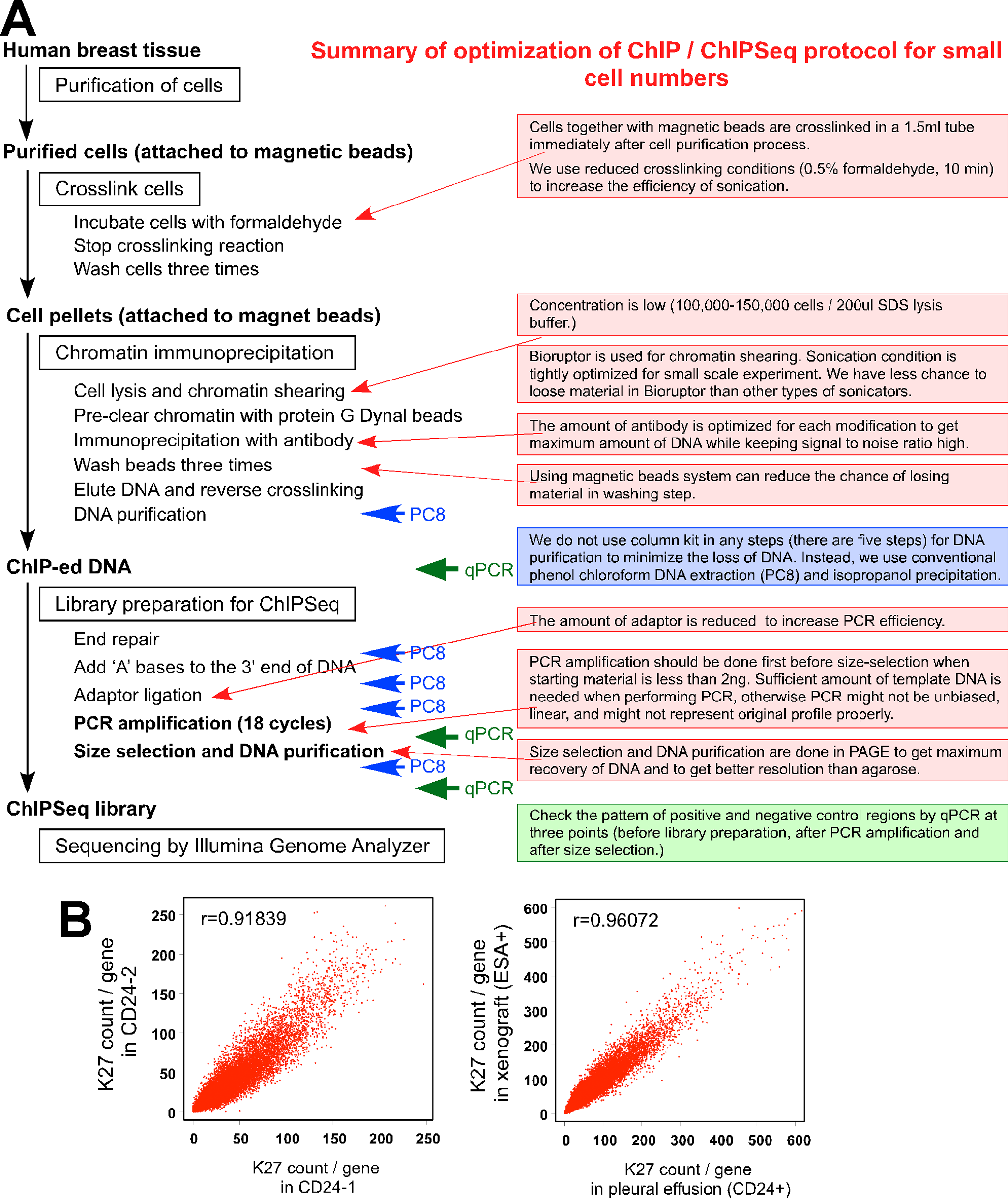

Supplement: Figure S1 — Comparison of small-scale and standard ChIP-Seq protocols. (A) Schematic outline of ChIP-Seq protocol. Critical steps that required optimization (red), DNA purification steps (blue), and quality control qPCR steps (green) are indicated. (B) Correlation of small-scale ChIP-Seq data between the same cell type from two different individual (left) and the same breast tumor purified two different ways (right). (1.43 MB TIF) [file pgen.1001369.s001.tif]

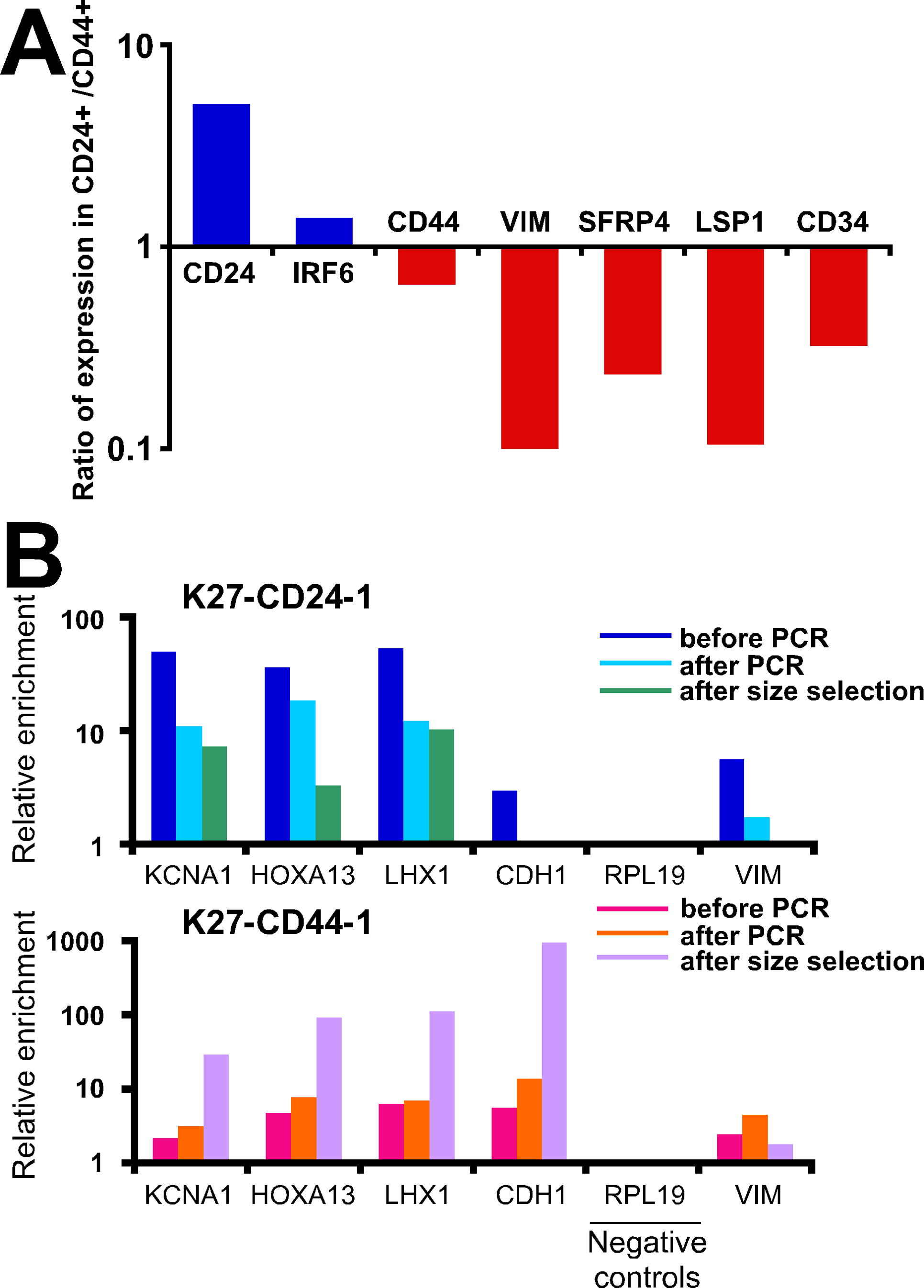

Supplement: Figure S2 — Confirmation of cell purity and histone methylation patterns. (A) We performed qRT-PCR for known markers of CD44+ progenitor (red) and CD24+ differentiated luminal epithelial (blue) cells to confirm the success of the purification procedure. Part (15%) of the fractionated cells was used for RNA preparation whereas the remaining fraction (85%) was used for the generation of ChIP-Seq libraries. (B) The quality of ChIPed DNA is evaluated by qPCR during ChIP-Seq library preparation before and after PCR amplification and after size selection steps. Bar plots depict representative results for K27 in CD24+ (top panel) and CD44+ (bottom panel) cells. Y-axis indicates enrichment relative to negative control (RPL19 promoter region); KCNA1, HOXA13, and LHX1 are putative K27-enriched genes, whereas CDH1 and VIM are CD24+ and CD44+ cell type–specific genes, respectively. (0.31 MB TIF) [file pgen.1001369.s002.tif]

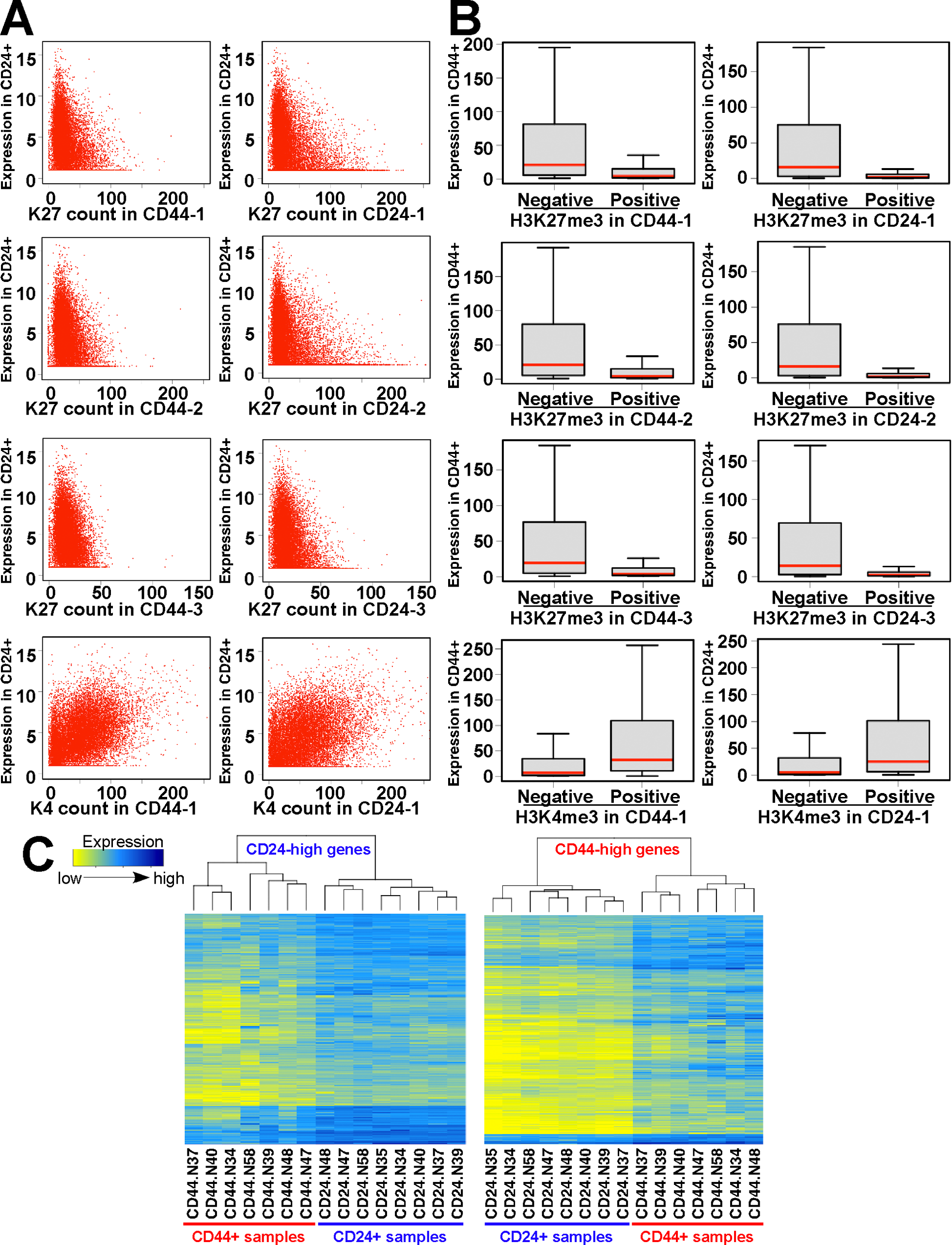

Supplement: Figure S3 — Correlation between histone modification and gene expression patterns in each sample. (A) Scatter plots comparing the level of histone modification and the level of gene expression. Each dot represents a gene. X-axis indicates mapped read counts around promoter region for the indicated histone mark and Y-axis indicates the median expression of the corresponding gene in the indicated libraries. (B) H3K27me3 and H3K4me3 states around gene promoter regions negatively and positively correlate with corresponding gene expression levels, respectively. (C) SAGE-Seq analysis of CD44+ progenitor-enriched and CD24+ luminal epithelial cells. Dendograms depicting the relatedness of the indicated samples. Hierarchical clustering was applied to SAGE-Seq data of selected cell type–specifically expressed genes. Heatmaps show consistently differentially expressed genes between CD24+ and CD44+ cells. We selected 435 genes consistently highly expressed in CD24+ cells and 656 genes consistently highly expressed in CD44+ cells. (1.63 MB TIF) [file pgen.1001369.s003.tif]

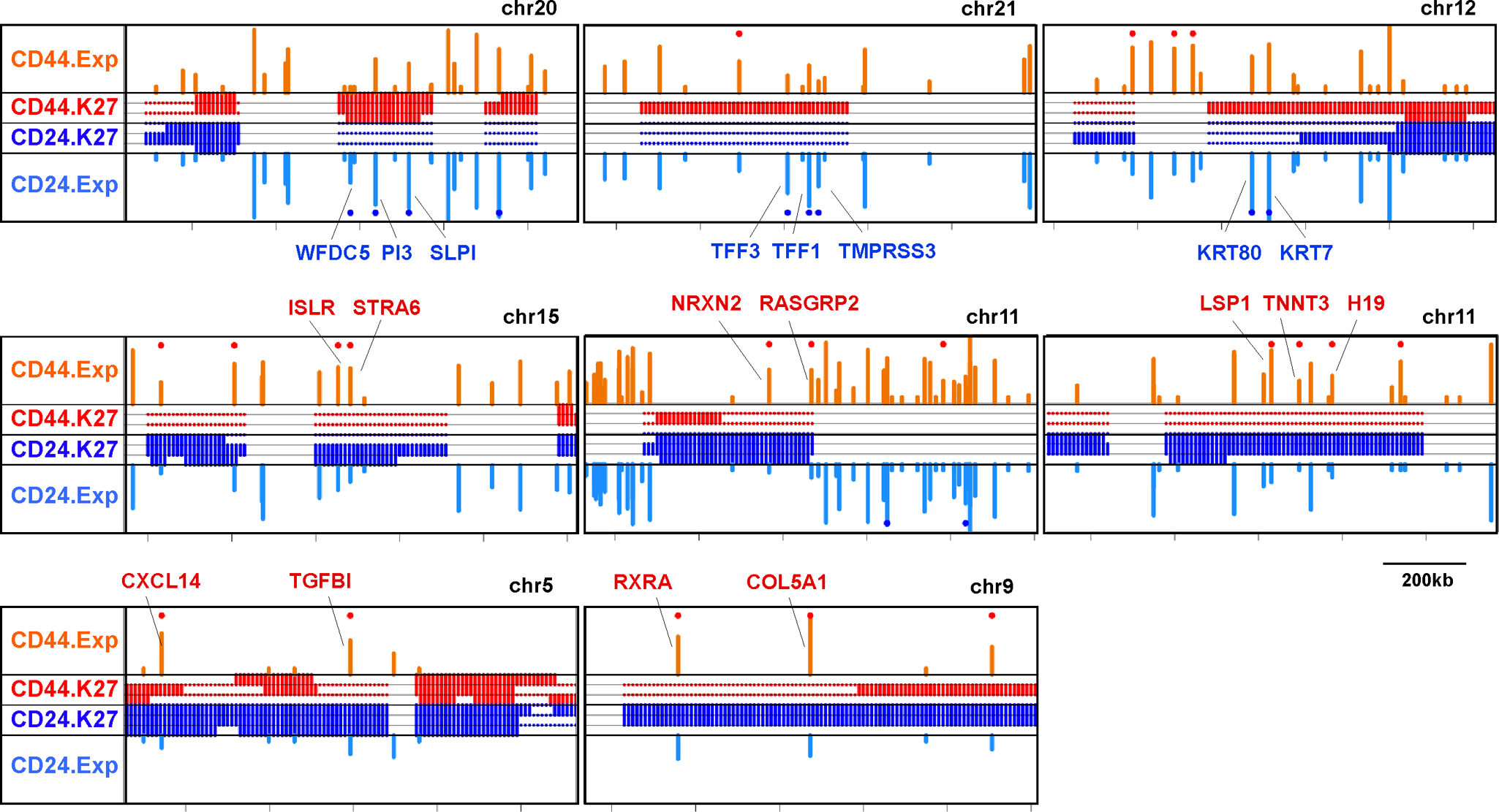

Supplement: Figure S4 — Examples of genes contained in K27 blocs. Patterns of K27 enrichment and gene expression are mutually exclusive. Examples showing clusters of genes located in K27 blocs and potentially silenced by this modification. Data was analyzed using SICER algorithm [28] using 10kb as window size. Significantly enriched regions and gene expression levels are plotted as colored lines across chromosome position. Red and blue lines represent K27 enrichment in CD44+ and CD24+ cell libraries from three different individuals, respectively. Orange and light blue: median expression in CD44+ and CD24+ samples, respectively. The height of line indicates the level of expression of corresponding gene. Red and blue dots indicate genes highly expressed (>2-fold difference) in CD44+ and CD24+ cells, respectively. (0.69 MB TIF) [file pgen.1001369.s004.tif]

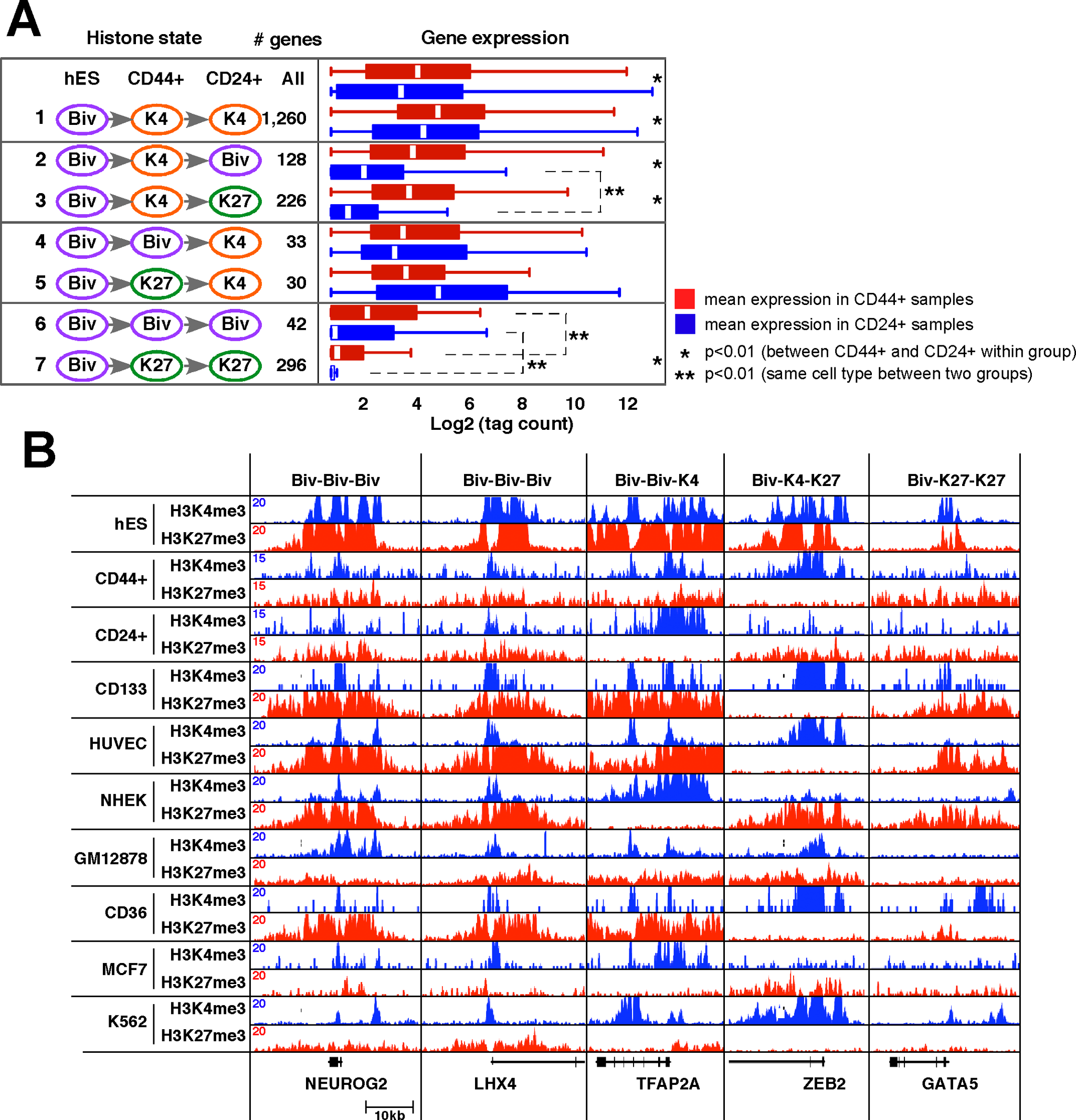

Supplement: Figure S5 — Chromatin states for “Biv-Biv-Biv” and “Biv-K27-K27” genes in different cell types. (A) Mean expression of genes (right bar graph) with the indicated chromatin pattern (left panel) in CD24+ and CD44+ samples. Wilcoxon rank sum test was performed to identify significant differences between CD44+ and CD24+ samples within the same group and between-groups. Asterisks indicate significant differences. (B) Representative examples of ChIP-Seq tag distribution in different cell types analyzed by the ENCODE project for several genes where we observed either bivalent or K27 mark in CD44+ and CD24+ cells. Total aligned tag count in each ChIP-Seq library was scaled to 10 million and tag counts averaged over a 10 bp window are shown in the Y-axis. NEUROG2, LHX4, and TFAP2A tend to keep K4 mark, whereas GATA5 tends to loose K4 mark in various cell types. (1.30 MB TIF) [file pgen.1001369.s005.tif]

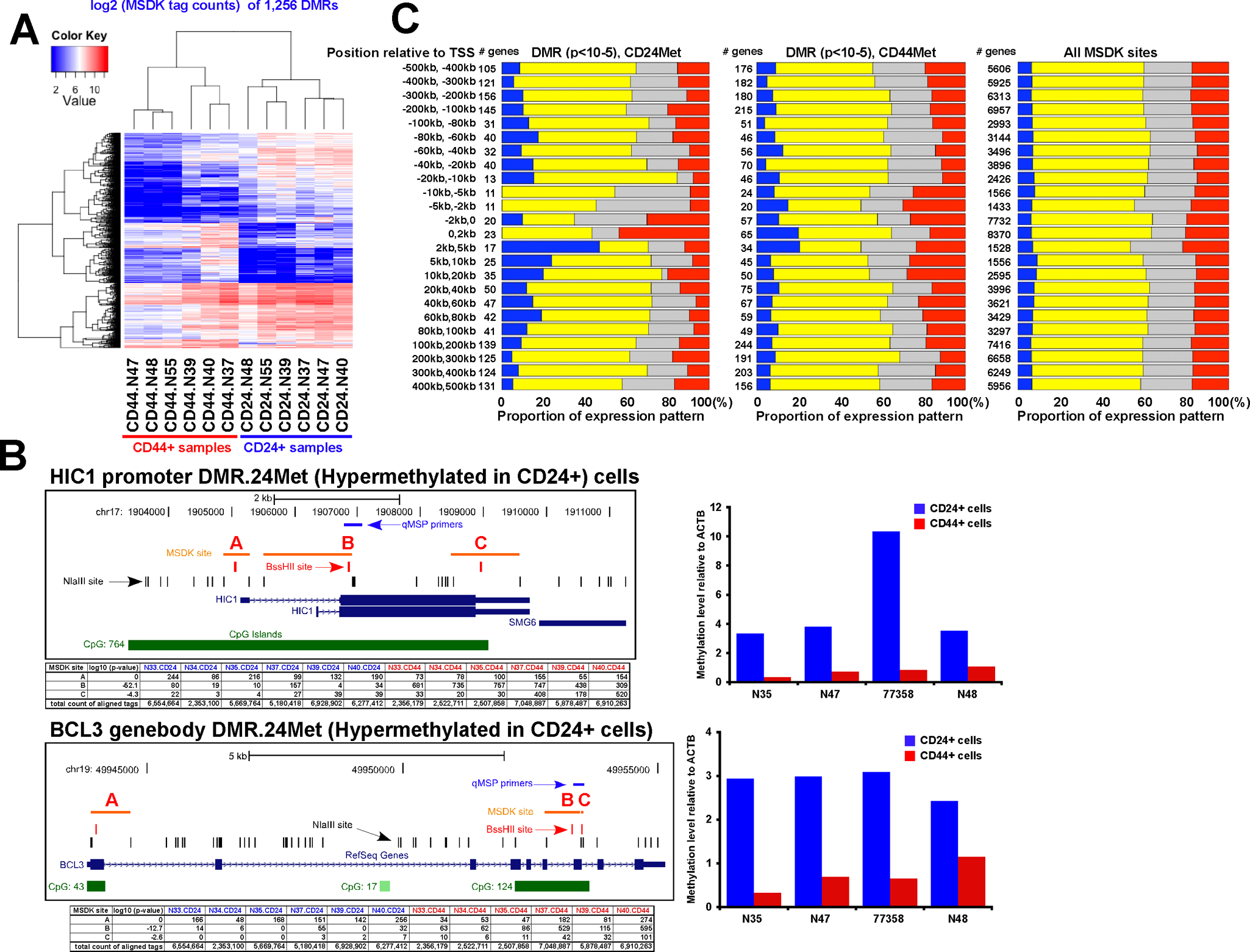

Supplement: Figure S6 — Examples of MSDK tag counts for selected genes with DMRs and their experimental validation and associations between DMRs and gene expression. (A) Heatmap depicting the clustering of samples based on tag counts for the 1,256 DMRs we identified. (B) Left: Schematic view of the indicated genomic region based on UCSC genome browser. Location of CpG islands, BssHII and NlaIII recognition sites, positions of primers used for qMSP are indicated. Right: Experimental validation of the indicated DMRs by qMSP. Cells purified from four independent individuals (N37, N38, 77358, and N48) are analyzed. Bars indicate methylation in CD44+ and CD24+ cells relative to ACTB. (C) Enrichment of DMRs in differentially expressed gene sets depending of the location of DMR relative to TSS. Observed/Expected ratios are shown for each combination. Expression pattern of each gene set is shown as bar chart. Genes in each gene set have indicated DMR (left panel:CD24-hypermethylaed DMR, middle: CD44-hypermethylaed DMR, right: any MSDK sites) in indicated location relative to their TSSs. red: genes highly expressed genes in CD24+ and CD44+ cells, respectively (≥2-fold change). Yellow and gray: genes with ≤2-fold difference between CD24+ and CD44+ cells and with low/no detectable expression in either cell type, respectively. (0.92 MB TIF) [file pgen.1001369.s006.tif]
