# Supplementary material for: Epigenetic Regulation of Cell Type–Specific Expression Patterns in the Human Mammary Epithelium
Source: PLoS Genet. 2011 Apr 21;7(4):e1001369. doi: 10.1371/journal.pgen.1001369 (PMC3080862; doi:10.1371/journal.pgen.1001369)
Supplement: Protocol S1 — Description of human tissue samples used for the generation of SAGE-Seq, ChIP-Seq, and MSDK-Seq libraries and number of aligned reads in each ChIP-Seq library and number of total tags in SAGE-Seq and MSDK-Seq libraries. (0.23 MB DOC) [file pgen.1001369.s007.doc]

**PROTOCOLS**

**Description of human tissue samples used for the generation of SAGE-Seq, ChIP-Seq, and MSDK-Seq libraries and number of aligned reads in each ChIP-Seq library and number of total tags in SAGE-Seq and MSDK-Seq libraries.**

| **SAGE-Seq** |  |  |  |  |  |
| --- | --- | --- | --- | --- | --- |
| **Tissue** | **Age** | **Ethnicity** | **Cell type** | **raw tag** | **aligned tag** |
| N33 | 19 | black | CD24+ | 9,618,916 | 6,401,701 |
| N34 | 27 | Black | CD44+ | 8,945,148 | 5,668,737 |
| N35 | 18 | black | CD24+ | 13,522,703 | 9,518,916 |
| N47 | 21 | black | CD24+ | 2,983,207 | 1,933,928 |
|  |  |  | CD44+ | 1,824,933 | 1,137,819 |
| N48 | 26 | caucasian | CD24+ | 1,800,069 | 1,098,424 |
|  |  |  | CD44+ | 1,045,874 | 695,300 |
| N37 | 24 | black | CD24+ | 4,861,324 | 3,295,244 |
|  |  |  | CD44+ | 4,170,428 | 2,696,351 |
| N39 | 32 | caucasian | CD24+ | 4,189,542 | 2,831,627 |
|  |  |  | CD44+ | 4,278,041 | 2,820,466 |
| N40 | 29 | black | CD24+ | 3,540,368 | 2,311,370 |
|  |  |  | CD44+ | 3,979,209 | 2,463,398 |
| N58 | 23 | black | CD24+ | 9,720,068 | 6,197,805 |
|  |  |  | CD44+ | 11,007,864 | 7,066,583 |
|  |  |  |  |  |  |
| **MSDK-Seq** |  |  |  |  |  |
| **Tissue** | **Age** | **Ethnicity** | **Cell type** | **raw tag** | **aligned tag** |
| N33 | 19 | black | CD24+ | 10,777,438 | 6,554,664 |
|  |  |  | CD44+ | 4,147,642 | 2,356,179 |
| N34 | 27 | black | CD24+ | 3,509,249 | 2,353,100 |
|  |  |  | CD44+ | 4,435,146 | 2,522,711 |
| N35 | 18 | black | CD24+ | 7,907,066 | 5,669,764 |
|  |  |  | CD44+ | 3,981,235 | 2,507,858 |
| N37 | 24 | black | CD24+ | 7,269,319 | 5,180,418 |
|  |  |  | CD44+ | 8,657,890 | 7,048,887 |
| N39 | 32 | caucasian | CD24+ | 8,556,003 | 6,928,902 |
|  |  |  | CD44+ | 8,259,680 | 5,878,487 |
| N40 | 29 | black | CD24+ | 7,491,800 | 6,277,412 |
|  |  |  | CD44+ | 8,335,235 | 6,910,263 |
|  |  |  |  |  |  |
| **ChIP-Seq (H3K27me3)** | |  |  |  |  |
| **Tissue** | **Age** | **Ethnicity** | **Cell type** | **raw tag** | **aligned tag** |
| N66 | 21 | black | CD24+ | 17,669,447 | 10,633,724 |
|  |  |  | CD44+ | 15,611,890 | 8,562,021 |
| N74 | 20 | black | CD24+ | 16,760,143 | 9,086,702 |
|  |  |  | CD44+ | 14,973,538 | 8,147,052 |
| N60 | 17 | black | CD24+ | 15,703,801 | 5,556,015 |
|  |  |  | CD44+ | 13,447,626 | 6,172,052 |
|  |  |  |  |  |  |
| **ChIP-Seq (H3K4me3)** | |  |  |  |  |
| Tissue | **Age** | **Ethnicity** | **Cell type** | **raw tag** | **aligned tag** |
| N66 | 21 | black | CD24+ | 13,824,008 | 7,948,684 |
|  |  |  | CD44+ | 12,819,455 | 7,143,074 |

**ChIP and ChIP-Seq experiment**

We optimized each step of the ChIP protocol to minimize the loss of material, and also tried several different procedures for ChIP-Seq experiments to see which option is better for this purpose. We found three critical steps required for good results: 1) Appropriate ratio of cell numbers and the amount of antibodies, 2) stringent sonication conditions, and 3) the order of PCR step in preparing ChIP-Seq libraries (Figure S1). Using the optimized ChIP-Seq protocol for small amount of DNA, we were able to perform ChIP-Seq experiment for histone modification from 1ng ChIP-ed DNA as starting material. To validate our optimized small-scale protocol we first analyzed the genome-wide H3K27 profiles of MCF7 cells using our small-scale and standard protocols.

Detailed optimized small-scale ChIP-Seq protocol: Following cell purification, part (15%) of the fractionated cells was used for RNA preparation to check their purity by qRT-PCR for several known cell type-specific markers. The remaining fraction (85%), 100,000-300,000 cells (number varies depending on cell fraction and tissue sample), was washed with 1ml DMEM/F12 medium (w/o serum) and re-suspended in 1ml DMEM/F12 (w/o serum). Cells were still attached to beads at this point, however, instead of capturing using magnet, we used centrifuge (at 3,000rpm for 2min) for every washing steps after this point. Cells were immediately cross-linked with formaldehyde (final concentration is 0.5%) at room temperature for 10 minutes. The reaction was stopped by incubation with glycin (1.25 M) for 5 min. and cells were washed three times [once with 1ml PBS+BSA (5mg/ml), once with 1ml PBS, and once with 0.2ml PBS+PIs (protease inhibitors: Complete mini, Roche)]. Cell pellets can be stored at –80^o^C for several days. 100,000-150,000 cells were re-suspended in 200ul SDS lysis buffer (1% SDS, 10 mM EDTA, 50 mM Tris-HCl pH 8.1, and protease inhibitors) and incubated on ice for 10 min. Higher cell numbers require higher volume and split into several tubes to keep the same cell-to-buffer ratio. Cell lysates were then sonicated using a Bioruptor (Diagenode) for 30 seconds at the maximum setting sixteen times with 30 seconds intervals. Magnetic beads were still present in the cell lysates during sonication. The sonicated lysates were centrifuged for 15 min., the supernatant was transferred to 20 μl pre-washed protein G magnetic beads (Invitrogen), and incubated at 4^o^C for 30 min. Antibodies, 2 μg of anti-H3K27me3 antibody (Millipore 07-449) or 0.2 μg of anti-H3K4me3 antibody (Abcam ab8580), were pre-incubated with 20 μl of protein G magnetic beads (Invitrogen) at 4^o^C for 2 hours before immunoprecipitation. 60 μl of the pre-cleared sheared chromatin (corresponding to 30,000-45,000 cells) was diluted with 540 μl of dilution buffer (1% Triton, 2 mM EDTA, 150 mM NaCl, 20 mM Tris-HCl pH 8.1) and incubated with the antibody-coated protein G magnetic beads at 4^o^C for 8 hours. 10μl of the pre-cleared sheared chromatin was saved as an input sample. The immunoprecipitates were sequentially washed (using magnetic stand) once with 1ml low salt wash buffer (0.1% SDS, 1% Triton X-100, 2 mM EDTA, 20 mM Tris-HCl pH 8.1, 150 mM NaCl), once with 1ml high salt wash buffer (0.1% SDS, 1% Triton X-100, 2 mM EDTA, 20 mM Tris-HCl pH 8.1, 500 mM NaCl), once with 1ml LiCl immune complex wash buffer (0.25 M LiCl, 1% IGEPAL-CA630, 1% deoxycholic acid, 1 mM EDTA, 10 mM Tris pH 8.1). Tubes were incubated on rotator for 5 min at 4^o^C between every wash. Then they were washed twice with 1ml TE. Samples were re-suspended in 100μl of 1%SDS and 0.1M NaHCO3, and incubated at room for 30 min. with occasional vortexing. 100μl of this solution was added to the input samples as well. Samples were incubated at 65^o^C for 4 hours to reverse crosslinking, followed by RNase A and proteinase K treatment for 1 hour. The recovered DNA was purified using PC8 (phenol-chloroform-isoamyl alcohol saturated with TE pH 8.0) extraction and isopropanol precipitation, and resuspended in 30μl of LoTE (0.1xTE). ChIP-ed DNA was end-repaired by END-It DNA End Repair Kit (Epicentre), and purified by PC8 extraction and isopropanol precipitation and resuspended in 34ul of LoTE. ‘A’ base was added to 3’-ends by Klenow fragment. DNA was purified by PC8 extraction and isopropanol precipitation and resuspended in 6 μl of LOTE and 1:10 diluted Adaptor oligo mix (ChIP-Seq Sample Preparation Kit, Illumina), ligated using T4 DNA ligase HC (Invitrogen), and subjected to PCR amplification (18 cycles) using specific primers provided in the kit (Illumina). Amplified DNA was tested by qPCR for several known markers. Fragments ranging from 150-300 bp were isolated from 8% polyacrylamide gel as described previously [2]. Purified DNA was again tested by qPCR for several known markers and then sequenced by Illumina Genome Analyzer.

**Comparison of small-scale and standard-scale ChIP-Seq experiment**

9x10^4^ and 3.4x10^6^ MCF-7 cells were used for small and large-scale experiment, respectively. “Broad ChIP Protocol for Full REMC” used by the epigenomics roadmap project was considered as standard protocol and modified for small-scale experiments including the amount of antibody and sonication conditions. Specifically, in standard scale experiment, 3.4x10^6^ cells were re-suspended in 340 μl SDS lysis buffer and subjected to sonication in Bioruptor for 20 cycles of 30 sec at high power and 30 sec off. Supernatant was split into two tubes and each was incubated with 2 μg of H3K27 antibody in ChIP dilution buffer overnight. In small-scale experiment, 9x10^4^ cells were re-suspended in 200 μl SDS lysis buffer and sonicated in Bioruptor for 16 cycles of 30 sec at high power and 30 sec off. Sheared chromatin was split into three tubes and each was incubated with 2 μg of H3K27 antibody in ChIP dilution buffer overnight.

Comparison of ChIP-Seq data quality obtained using the two methods:

| **MCF7-K27** | **raw reads** | **aligned reads** | **% aligned** | **duplicate reads** | **% duplicate** |
| --- | --- | --- | --- | --- | --- |
| Small-scale | 20,663,238 | 14,629,180 | 70.80 | 568,256 | 3.88 |
| Standard-scale | 20,944,677 | 13,805,185 | 65.91 | 441,169 | 3.20 |

**Quantitative methylation-specific PCR (qMSP)**

qMSP was performed essentially as described previously [3]. Primers used for HIC1 were 5’-GTGTAGAACGTTTTTTTTCGCGC-3’ (Forward) and 5’-CCACCAAAAACTTAAAATAAACGCTACTAACCG-3’ (Reverse). Primers for BCL3 were 5’-GTAGTTTCGTTTTGTATTTAGCGTTCGGTC-3’ (Forward) and 5’-GCCACCATAAACGACGTATCG-3’(Reverse).

**ChIP-Seq data analysis**

We used the SICER algorithm [1], a spatial clustering approach for the identification of ChIP-enriched regions. This algorithm is especially useful for the analysis of diffuse enrichment covering extended genomic regions such as H3K27me3 modifications. Only uniquely mapped reads were used and all libraries were preprocessed to filter out redundant reads to minimize potential PCR bias. Reads were shifted by +/- 75bp to the center of DNA fragment depending on their strands. We applied SICER using default parameters (window size=200bp, effective human genome size=74.3%, window P-value=0.2). The gap size was g=1 (200bp) for H3K4me3 and g=3 (600bp) for H3K27me3 following the authors recommendation. We chose FDR<0.001 as cut off for the identification of significantly enriched islands. To define enriched genes, we analyzed the promoter regions of genes for overlap with histone-enriched islands. We classified each gene as "K27-enriched and positive" if there was a significant K27 enriched island within +/-5kb from its transcription start site (TSS), and if there was no island in the region, the gene was classified as "K27-not enriched and negative". Similarly, we classified each gene as "K4-enriched and positive" if a significant K4-enriched island overlapped with its TSS.

To analyze association between histone enrichment and gene expression, we prepared four gene sets. All genes were classified into four groups based on their gene expression patterns. Differentially expressed genes between CD44+ and CD24+ cells were selected by performing pair-wise comparisons using Z-statistics. We compared all 56 combinations between CD24+ and CD44+, and then we selected genes that showed significant difference (fold change >1.8 and z-score >2.33) in more than 40 out of all possible combinations. Finally, we obtained 435 genes consistently highly expressed in CD44+ cells (CD44-high genes) and 656 genes highly expressed in CD24+ cells (CD24-high genes) (Figure S5). These genes were functionally distinct and consistent with our previous findings [3]. Genes with low/no expression levels were defined by low expression proportion (<1x10^-6^) in both of cell types. The rest of the genes were classified as “no difference” genes.

Definition of bivalent states. We downloaded aligned ChIP-Seq data for histone H3K4me3 and H3K27me3 modifications in H1-hESC (Human Embryonic Stem Cells), GM12878 (lymphoblastoid), HUVEC (umbilical vein endothelial cell), K562 (chronic myelogenous leukemia), and NHEK (epidermal keratinocytes) from the ENCODE Data Coordination Center at UCSC (<http://genome.ucsc.edu/ENCODE>). Aligned bed files for CD36+ and CD133+ cells were downloaded from GEO Data sets (GSE12646). All public ChIP-Seq data was processed using our data analysis pipeline. We assigned each gene into one of the following four categories based on the combination of promoter histone modification states as described above. Bivalent: positive for both K4 and K27, K4: positive only for K4, K27: positive for K27 only, N: negative for both K4 and K27.

Definition of K27 blocs. For the definition and visualization of K27 blocs depicted in Figures 4 and S4, we used the SICER [1] algorithm setting 10kb as window size, g=3 (30kb) for gap size, and FDR=0.05 as threshold. We visualized genome-wide K27 distribution patterns by plotting only significantly enriched islands in 10kb bins. Next, we examined associations between the length of K27 blocs and the number of blocs, and the number of genes in blocs (Figure 4C). We examined overlaps (+/-5kb from TSS of genes) between K27 blocs (defined at various lengths) and differentially expressed genes, and calculated the fraction of differentially expressed genes in K27 blocs relative to all differentially expressed genes (Figure 4D).

**MSDK-Seq Data analysis**

Cluster generation and sequencing-by-synthesis on the Illumina Genome Analyser II (running SCS2.3 / IPAR, Pipeline 1.3.2). Each library was sequenced on a single lane of an 8-lane flow-cell. The sequence files were aligned against the hg18 reference genome using the ELAND extend using Illumina default parameters. Sorted and indexed files were compared against the location of NlaIII sites, adjacent to BssHII sites in the genome, to generate a tabular form of the data, which reflects the methylation status of each BssHII site in each sample.

We assume there are *n*-pairs of sequencing experiments. Each pair involves two tissue samples with two corresponding libraries, $L_{iA}$ and $L_{iB}$, where *i* = 1,…,*n* and *A*=`CD24’ and *B*=`CD44’. For our analysis here the outcomes are two total counts of tags mappable to our effective genome uniquely and exactly, $y_{iA}$ and $y_{iB}$, and two counts of tags which were mapped to a particular region of interest *R,* denoted $x_{iA}$ and $x_{iB}$, respectively. Obviously, for each alternative region R the analysis outlined below is repeated.

We compute for each $i=1,2,...,n$ and *a = A, B* the following statistic:

,

where is the ratio of the total numbers of mapped tags, *b* denotes the complement of *a* in , and is the (unique) solution for of the following equation:

.

.

The solution is found using the iterative Newton method (Kowalczyk et al., manuscripts in press and in preparation). In typical case only one of the two values
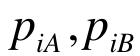
 is significant, i.e. <<1 while the other one is $\approx1$.

Now we choose the direction of the hypothesis to be tested and degree *d*, . If *a* = *A* = `CD24’ , then we will be testing the hypothesis that region *R* in at least *n-d* out of *n* libraries is more methylated than in corresponding libraries . This means that

,

for at least *n-d* indices *i*, where denotes the proportion of the total number of mappable reads in the library which fall into the region *R* for . The unobservable proportions are of central interest to us, and the goal of the analysis is to derive information about their relationships from the observed data.

Let us assume for convenience the indices are chosen in the descending order of , i.e. . We compute the following test statistic for the whole experiment, called the *Poisson margin* (Kowalczyk, et al., manuscripts in press and in preparation):

where the “small” exponent of the error factor has the bounds

.

In the particular case of *d* = 0 we have

,

where

;

for *d* =1 we get

,

where

.

As typical values of for significant peaks are < -100, and the differences are of the same order, so the correction term $\varepsilon$ can be practically neglected.

The statistic has been custom developed as a tight upper bound on the p-value for acceptance of the alternative hypothesis that for at least *n-d* out of *n* sample pairs *i* the (unobservable) proportions of the read mapping to the region *R* satisfy the relation. This is derived under the modeling assumption that for each library the count is an instance of a conditional random variable with Poisson distribution:

,

for *x* = 0, 1, 2, … and . This is a typical modeling assumption in NGS analysis. Note that in the whole analysis above we did not assume that total sizes of sequenced tags are equal, or approximately equal. The statistic is compensating for such differences seamlessly, in a principled way. It is quite important in the current case as the values of differ by significant factors, up to 3.6. More comprehensive treatment of the subject can be found in the forthcoming dedicated methods paper (Kowalczyk, et al., manuscript in preparation) available as a technical report upon request. The whole analysis as outlined above has been repeated for each of the 32,453 MSDK regions, for *d* = 0 and *d* = 1 and with *n* = 6. The whole analysis required < 5 minutes of cpu time on a standard laptop using a Matlab implementation.

We predicted 44,108 possible MSDK regions based on the location of BssHII and NlaIII sites in the human genome and observed actual MSDK tags for 32,453 MSDK regions. Out of total 32,453 observed MSDK regions, 48.5% were located within +/-5kb from RefSeq TSS, 63.4% were located within +/-20kb. 56.0% of all MSDK regions overlapped with CpG islands.

To examine associations between DMRs and differential gene expression (Figure 6A), we first prepared gene sets where genes have DMRs (blue: CD24Met (p<10^5), pink: CD44Met (p<10^5), black: All MSDK sites) in certain positions relative to TSS indicated in X-axis. We next examined the expression patterns of these genes using the following four categories (CD24-high and CD44-high: genes highly expressed in CD24+ and CD44+ cells, respectively (≥2-fold change), low in both genes: averaged tag count <5, no-difference genes: others, same as Figure 6B and S6). We plotted fraction of each expression category (in Y-axis) against position of DMR relative to TSS (X-axis). For example, in the left upper panel we observed that genes that have CD24Met-DMR in downstream regions show CD24-high (expression) pattern at higher frequency than control gene set, (genes that have “All MSDK sites”). Similar analysis was performed in Figure 6B. We prepared several gene sets where genes have promoter DMR (-5kb to 2kb from TSS, left upper panel) and gene-body DMR (2kb to end of transcript, right upper panel) applying different stringency criteria (-log10p-value: 2,5,10,20) for DMRs. We examined expression patterns using the same criteria as Figure 6A and categorized genes in each gene set into four groups (blue, red, yellow, and gray). We prepared control gene sets where genes have any MSDK sites in the indicated regions and we plotted these in the top row in Figure 6B. We also randomized the same number of MSDK sites as DMRs and performed the same analysis with results depicted in the lower panels of Figure 6B. We do not see specific gene expression patterns in randomized MSDK sites data.

To examine possible associations between DMRs and histone modifications (Figure 7A), we analyzed +/-5kb (for K27 mark) and +/-0kb (for K4 mark) regions around each MSDK site for overlap with histone enriched islands. Based on this analysis we assigned histone enrichment states to each MSDK site. To perform integrated analysis of MSDK-Seq, ChIP-Seq, and SAGE-Seq data (Figure 7B), we first prepared four gene sets (group A: CD44-high/ K27-, CD44-high/ K27+, CD24-high/ K27+ and CD24-high/ K27-) based on differential expression and K27 enrichment similar to what we did in Figure 3E. We also prepared four gene sets (group B: genes associated with CD24Met or CD44Met in gene body or promoter region) based on DMRs similar to what we did in Figure 6B. Then we examined if any of the gene sets in group B are enriched for any of the four gene sets in group A by performing chi-square test for each combination (total 4x4=16 combinations) and using genes associated with any MSDK site in gene body or promoter region (as we did in top row of Figure 6B) as background.

**GO term enrichment analysis,**

GO term enrichment analyses were performed using David bioinformatics resources [4], we used all RefSeq genes that we used for mapping as background for calculating enrichment.

**GeneGo functional analysis using Metacore**

**Enrichment analysis** in the ontologies of canonical pathway maps, protein functions and GeneGo process networks was performed in MetaCore data analysis suite as described [5].

**Evaluation of network topology**. Topology analysis gives information about how tightly the GeneGo Global Network nodes from explored gene list of interest are connected.

*Degree* is the average number of links (interactions) connected to a node (protein). Since our network is directed, the nodes are characterized by *IN* and *OUT-degree*, giving the average number of outgoing and incoming interactions.

The *Clustering coefficient* captures the degree connectivity between node’s neighbors. It is defined as: , where *n_i_* is the number of links among the *k_i_* neighbors of node *i.* As *k_i_(k_i_-1)/2* is the maximum number of such links, the clustering coefficient is a number between 0 and 1. The average clustering coefficient is obtained by averaging over the clustering coefficient of individual nodes. A network with high clustering coefficient is characterized by highly connected sub-graphs.

**Enrichment by protein classes**. Gene lists of interest were analyzed for relative enrichment with certain protein classes. The results were ranked by a p-Value. The p-Values were calculated using the basic formula for a hypergeometric distribution (listed below) where r is the number of object of particular protein class from the set of interest (gene list); R is the number of object in set of interest; n is the number of object of particular protein class in the whole GeneGo Global Network; N is the number of object in whole GeneGo Global Network.


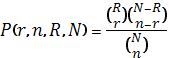


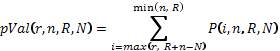


**Calculation of relative connectivity of proteins inside the subset (intra-connectivity) and between the set and the global interactome.** Gene lists (sets) were converted into proteins and then all protein lists were screened for the number of interactions with the global interactome - GeneGo Global Network (inter-connections) and within the protein lists (intra-connections). All proteins were divided onto 7 different functions (protein target classes): transcription factors, receptors, ligands, kinases, proteases, phosphatases, and metabolic enzymes. The expected number of interactions for a given protein with (for inter-connected)\within (for intra-connected) the protein list is determined as a fraction of the total number of its interactions in the GeneGo Global Network (global interactome) proportional to the size of the protein list. If the number of interactions with (for inter-connected)\within (for intra-connected) the protein list is larger than expected, the protein is considered as over-connected; if the number of interactions is lower than expected, the protein is considered as under-connected. The degree of over- and under-connectivity can be evaluated by z-score and p-value. p-values were calculated using the basic formula for a hypergeometric distribution listed above where r – number of proteins derived from current protein list that have interactions with given protein; R – total number of proteins in the GeneGo Global Network that have interactions with given protein; n – total number of proteins in given protein list; N - total number of proteins in the GeneGo Global Network.


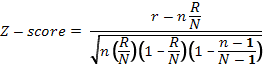


**SUPPORTING REFERENCES**

1. Zang C, Schones DE, Zeng C, Cui K, Zhao K, et al. (2009) A clustering approach for identification of enriched domains from histone modification ChIP-Seq data. Bioinformatics 25: 1952-1958.

2. Hu M, Yao J, Polyak K (2006) Methylation-Specific Digital Karyotyping. Nature Prot 1: 1-16.

3. Bloushtain-Qimron N, Yao J, Snyder EL, Shipitsin M, Campbell LL, et al. (2008) Cell type-specific DNA methylation patterns in the human breast. Proc Natl Acad Sci U S A 105: 14076-14081.

4. Huang da W, Sherman BT, Lempicki RA (2009) Systematic and integrative analysis of large gene lists using DAVID bioinformatics resources. Nat Protoc 4: 44-57.

5. Nikolsky Y, Kirillov E, Zuev R, Rakhmatulin E, Nikolskaya T (2009) Functional analysis of OMICs data and small molecule compounds in an integrated "knowledge-based" platform. Methods Mol Biol 563: 177-196.
